# Simultaneous Identification of *EGFR,* *KRAS,* *ERBB2,* and *TP53* Mutations in Patients with Non-Small Cell Lung Cancer by Machine Learning-Derived Three-Dimensional Radiomics

**DOI:** 10.3390/cancers13081814

**Published:** 2021-04-10

**Authors:** Tiening Zhang, Zhihan Xu, Guixue Liu, Beibei Jiang, Geertruida H. de Bock, Harry J. M. Groen, Rozemarijn Vliegenthart, Xueqian Xie

**Affiliations:** 1 Department of Radiation Oncology, Shanghai General Hospital, Shanghai Jiao Tong University School of Medicine, Haining Rd.100, Shanghai 200080, China; tiening69@126.com; 2 Siemens Healthineers Ltd., Zhouzhu Rd.278, Shanghai 200120, China; zhihan.xu@siemens-healthineers.com; 3 Department of Radiology, Shanghai General Hospital, Shanghai Jiao Tong University School of Medicine, Haining Rd.100, Shanghai 200080, China; xuemo6688@hotmail.com (G.L.); jennifer.chiang@hotmail.com (B.J.); 4 Department of Epidemiology, University Medical Center Groningen, University of Groningen, Hanzeplein 1, 9713 GZ Groningen, The Netherlands; g.h.de.bock@umcg.nl; 5 Department of Lung Diseases, University Medical Center Groningen, University of Groningen, Hanzeplein 1, 9700 RB Groningen, The Netherlands; h.j.m.groen@umcg.nl; 6 Department of Radiology, University Medical Center Groningen, University of Groningen, Hanzeplein 1, 9700 RB Groningen, The Netherlands; r.vliegenthart@umcg.nl

**Keywords:** radiomics, next-generation sequencing (NGS), non-small cell lung cancer (NSCLC), screening, computed tomography

## Abstract

**Simple Summary:**

Multiple genetic mutations are associated with the outcomes of patients with non-small cell lung cancer (NSCLC) after using tyrosine kinase inhibitor, but the cost for detecting multiple genetic mutations is high. Few studies have investigated whether multiple genetic mutations can be simultaneously detected based on image features in patients with NSCLC. We developed a machine learning-derived radiomics approach that can simultaneously discriminate the presence of *EGFR*, *KRAS*, *ERBB2*, and *TP53* mutations on CT images in patients with NSCLC. These findings suggest that machine learning-derived radiomics may become a noninvasive and low-cost method to screen for multiple genetic mutations in patients with NSCLC before using next-generation sequencing tests, which can help to improve individualized targeted therapies.

**Abstract:**

Purpose: To develop a machine learning-derived radiomics approach to simultaneously discriminate epidermal growth factor receptor (*EGFR*), Kirsten rat sarcoma viral oncogene (*KRAS*), Erb-B2 receptor tyrosine kinase 2 (*ERBB2*), and tumor protein 53 (*TP53*) genetic mutations in patients with non-small cell lung cancer (NSCLC). Methods: This study included consecutive patients from April 2018 to June 2020 who had histologically confirmed NSCLC, and underwent pre-surgical contrast-enhanced CT and post-surgical next-generation sequencing (NGS) tests to determine the presence of *EGFR*, *KRAS*, *ERBB2*, and *TP53* mutations. A dedicated radiomics analysis package extracted 1672 radiomic features in three dimensions. Discriminative models were established using the least absolute shrinkage and selection operator to determine the presence of *EGFR*, *KRAS*, *ERBB2*, and *TP53* mutations, based on radiomic features and relevant clinical factors. Results: In 134 patients (63.6 ± 8.9 years), the 20 most relevant radiomic features (13 for *KRAS*) to mutations were selected to construct models. The areas under the curve (AUCs) of the combined model (radiomic features and relevant clinical factors) for discriminating *EGFR, KRAS, ERBB2,* and *TP53* mutations were 0.78 (95% CI: 0.70–0.86), 0.81 (0.69–0.93), 0.87 (0.78–0.95), and 0.84 (0.78–0.91), respectively. In particular, the specificity to exclude *EGFR* mutations was 0.96 (0.87–0.99). The sensitivity to determine *KRAS*, *ERBB2*, and *TP53* mutations ranged from 0.82 (0.69–90) to 0.92 (0.62–0.99). Conclusions: Machine learning-derived 3D radiomics can simultaneously discriminate the presence of *EGFR*, *KRAS*, *ERBB2*, and *TP53* mutations in patients with NSCLC. This noninvasive and low-cost approach may be helpful in screening patients before invasive sampling and NGS testing.

## 1. Introduction

Lung cancer is responsible for 11.6% of de novo malignancies and 18.4% of cancer-related deaths in 2018 [1]. Over the past 15 years, the treatment of non-small cell lung cancer (NSCLC) has changed dramatically with the introduction of tumor genomic profiling and targeted therapy [2]. Several genetic mutations were identified in patients with NSCLC. The prevalence of mutations varies with ethnicity [3]. Epidermal growth factor receptor (*EGFR*) mutations exist in 40–60% of pulmonary adenocarcinoma in the Asian population, while only 7–10% in the European population [4]. Kirsten rat sarcoma viral oncogene (*KRAS*) mutation accounts for approximately 25% of patients with NSCLC [5]. A total of 27% of the Caucasians had the *KRAS* mutation, significantly higher than 17% of African Americans [6]. The over-expression of Erb-B2 receptor tyrosine kinase 2 (*ERBB2*) was observed in 2.4–38% of NSCLC cases [7,8]. Tumor suppressor protein 53 (*TP53*) gene mutation can be found in 35–60% of patients with NSCLC [9]. These gene mutations are associated with the prognosis of patients with NSCLC after receiving tyrosine kinase inhibitor (TKI) therapy and may confer resistance to TKI [10]. For example, *EGFR* is the main actionable target in patients with NSCLC [11]; a recent trial showed that sotorasib can be used against NSCLC harboring *KRAS* mutation [12].

Detection of multiple genetic alterations in patients with lung cancer is crucial to decide the applicability of targeted therapy. Next-generation sequencing (NGS), a high-throughput genetic sequencing method, allows for simultaneous and rapid detection of multiple tumor mutations [13,14]. NGS achieved an accuracy of 99.1% for detecting *EGFR* mutation in patients with advanced lung adenocarcinoma, compared with the traditional Sanger sequencing method [15]. Thus, many medical centers used NGS in clinical practice [16]. However, the current clinical practice for NGS involves invasive biopsy or surgical resection, which is associated with high cost and patient discomfort. Intra-tumor heterogeneity, which leads to the heterogeneous molecular sampling results, reduces the accuracy of identifying potential genetic mutations [17]. Furthermore, in some areas, the clinical implementation of NGS is still poor. A comprehensive and noninvasive approach will help to screen candidate patients for invasive sampling and NGS testing.

Radiomics, a subfield of machine learning, is a promising noninvasive approach to assess genetic mutations in lung cancer. Radiomics extracts and analyzes a large number of advanced quantitative image features with high throughput. This approach can be used to determine the molecular type of lung tumors based on the phenotypic appearance in computed tomography (CT) [18]. Several studies have reported encouraging results in discriminating *EGFR* mutation using radiomics [19,20]. For example, Jia et al. built a random forest classifier to identify *EGFR* mutation and reached an area under the receiver operating characteristics curve (AUC) of 0.802 [21]. Pinheiro et al. found that radiomic features can discriminate *EGFR* mutation with an AUC of 0.75, but did not find a radiomic feature correlated to *KRAS* mutation [22]. To our knowledge, few studies have investigated whether multiple genetic mutations can be simultaneously detected based on image features in patients with NSCLC. Therefore, this study aimed to develop a machine learning-derived radiomics approach to discriminate the presence of *EGFR*, *KRAS*, *ERBB2*, and *TP53* mutations on CT images in patients with NSCLC.

## 2. Methods

### 2.1. Study Population

This study retrospectively included consecutive patients with NSCLC who visited our institute from April 2018 to June 2020. The inclusion criteria were as follows: (1) surgically resected tumor sample tissues; (2) patients with NSCLC confirmed by hematoxylin-eosin and immunohistochemistry staining; (3) post-surgical NGS test proved the mutation status of *EGFR*, *KRAS*, *ERBB2*, and *TP53*; (4) thin-slice contrast-enhanced chest CT (slice thickness ≤ 1 mm) performed prior to tumor resection; (5) interval between CT scanning and tumor resection < 1 month. The exclusion criteria were: (1) non-contrast CT examination; (2) low-quality CT images affected by image artifacts; (3) indistinguishable tumor edge, caused by adjacent obstructive pneumonia, atelectasis, and mediastinal adhesions, etc. The collected clinical factors were age at diagnosis, sex, cTNM stage, smoking status, and tumor location. The cTNM stage categorizes the extent of the tumor during imaging examination before any treatment. The cTNM stage was determined by whole-body CT except the lower extremities or whole-body PET-CT.

**Figure 1:**
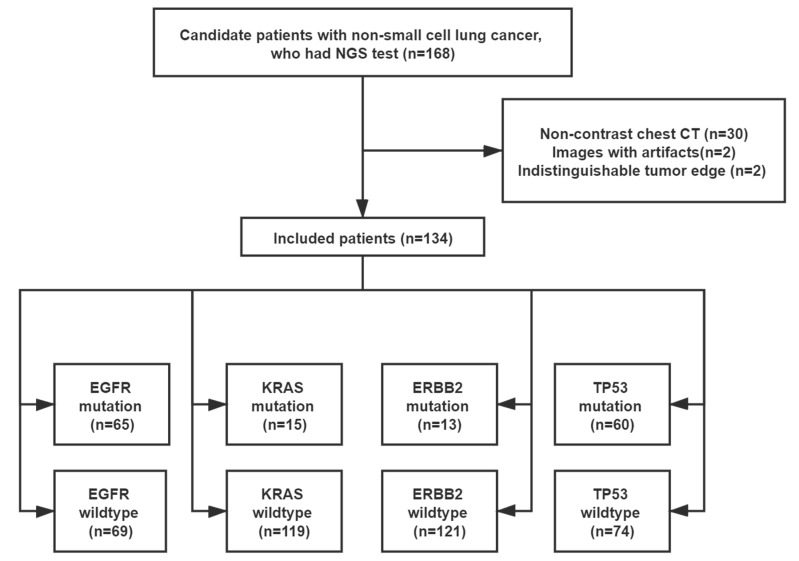
Patient inclusion flowchart. NGS = next generation sequencing; *EGFR* = epidermal growth factor receptor; *KRAS* = Kirsten rat sarcoma viral oncogene; *ERBB2* = Erb-B2 receptor tyrosine kinase 2; *TP53* = tumor protein 53.

The local Institutional Review Board approved this retrospective study (No. SGH-2018-56) and waived the requirement for patient informed consent. The patient selection flowchart is shown in Figure 1.

### 2.2. NGS

In this study, a Clinical Laboratory Improvement Amendments (CLIA)-certified testing center (Burning Rock Biotech, Guangzhou, China) performed deoxyribonucleic acid (DNA) processing and subsequent NGS procedures for adequate formalin-fixed and paraffin-embedded tumor sections to detect somatic genetic mutations. In brief, a minimum of 50 ng of DNA isolated from the tumor tissue was processed for NGS library construction and profiled using the capture-based targeted sequencing panels targeting multiple genes. NGS was performed by using an ultra-deep (20,000×) 168-gene panel named LungPlasma (Burning Rock Biotech, Guangzhou) [23]. Sequencing panels were selected based on the patients’ clinical characteristics and financial situation. The panels interrogated the whole exons and critical intronic regions of the actionable genes including *EGFR*, *KRAS*, *ERBB2*, and *TP53* in this study.

### 2.3. CT Image Acquisition

All included patients underwent contrast-enhanced chest CT scans using two CT scanners (Somatom Force, Siemens Healthineers, Erlangen, Germany; Revolution CT, GE Healthcare, Milwaukee, WI, USA). A total of 60–80 mL of contrast medium (Iopamiro 300, Bracco, Milan, Italy) was injected at 4 mL/s. The reconstructed slice thickness was 0.6 mm and 0.625 mm, respectively. Table S1 presents the detailed acquisition protocol and reconstruction parameters.

### 2.4. Lesion Segmentation and Extraction of Radiomic Features

**Figure 2:**
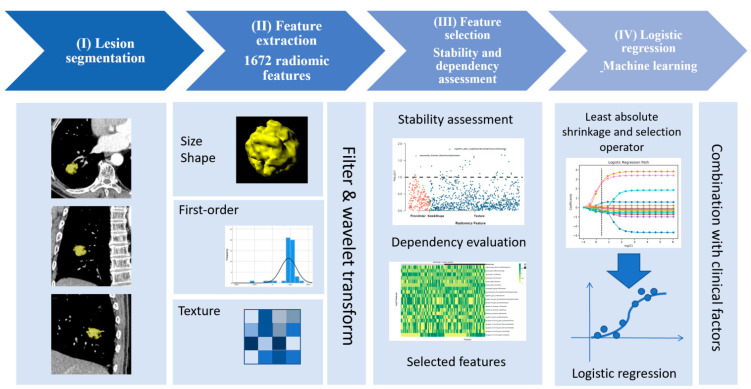
Pipeline step diagram of machine learning-derived radiomics.

Figure 2 shows the radiomics analysis pipeline steps. One radiologist with 18 years of experience in diagnostic imaging, who was blinded to the results of the NGS test, performed semi-automated three-dimensional (3D) tumor segmentation, using a radiomics analysis software package (Radiomics 1.0.9a, Siemens Healthineers) on a research platform (SyngoVia VB10b, Research Frontier, Siemens Healthineers). This radiomics analysis package extracts radiomics features based on the Pyradiomics library [24], in conformance with the Image Biomarker Standardization Initiative [25]. After finding the lesion and clicking on it, the software automatically segments the tumor edge and extracts 1672 radiomic features. These features comprise first-order (HU stats), shape, and texture features. The first-order feature describes the intensity distribution of CT values in the volume of interest by common basic measures, such as mean, range, and standard deviation [24]. The texture features comprise the following five categories: (1) gray-level co-occurrence matrix; (2) gray-level difference matrix; (3) gray-level run-length matrix; (4) gray-level size-zone matrix; (5) neighborhood gray-tone difference matrix. These features are analyzed by nine filters and eight wavelet transformations in high dimensions. Details on the principle of feature algorithms are found in the supplementary materials.

### 2.5. Selection of Radiomic Features

To assess the stability of feature extraction, two observers with 5 and 18 years of experience in radiology independently evaluated 50 randomly selected patients. Spearman’s rank correlation coefficient between the two feature extracting procedures was calculated to indicate the feature stability [26]. The features with a Spearman’s *r* > 0.8 were considered stable for the subsequent analysis. Then, according to the F-statistic test in one-way analysis of variance (ANOVA), the most correlated features with the presence of genetic mutations were selected. In radiomics studies, this method is commonly used for univariant feature selection by estimating the degree of linear dependency between features and labels (mutations in our study) [27,28]. The top 20 significant features to the presence of mutations were eventually selected to establish the discriminative models.

### 2.6. Model Development

In this study, the one-vs-all strategy, which exhibited great interpretability and fitted one classifier per class, was implemented to achieve the aim of the multi-classification task to identify four different mutation types [29]. First, we established four discriminative models based only on radiomic features (radiomics models) to determine the presence of *EGFR*, *KRAS*, *ERBB2*, and *TP53* mutations, using penalized multivariate logistic regression with 5-fold cross-validation. The least absolute shrinkage and selection operator (LASSO) was implemented for imposing a penalty to the logistic model with excessive features, so that the coefficient of noncontributing features shrank to zero. LASSO logistic regression, as a machine learning algorithm, is commonly used to select contributing features in radiomics research [30].

Second, we built four other discriminative models (combined models), each of which was based on the combination of radiomic features and clinical factors, to predict the existence of *EGFR*, *KRAS*, *ERBB2*, and *TP53* mutations. The Wilcox rank-sum test was used to select significantly relevant clinical factors associated with the presence of a genetic mutation. Then, multivariate logistic models combining radiomic features and significant clinical factors were established to discriminate the presence of mutations.

### 2.7. Statistical Analysis

A one-sample Kolmogorov–Smirnov test was applied for the normality test of continuous variables. The Fisher exact test or Chi-square test was used to compare categorical variables, and the independent Student *t*-test or Mann–Whitney U test for continuous variables. The discrimination performance of models was evaluated by the receiver operating characteristics (ROC) curve. The cutoff value was obtained by using the maximum likelihood ratio on the ROC curve. Sensitivity, specificity, and accuracy were calculated based on these cutoff values. DeLong’s test was used to compare the diagnostic performance between the radiomics model and combined model for each of the four genetic mutations.

The Python Scikit-learn package (Python v3.7, Scikit-learn v0.22, https://scikit-learn.org, accessed on 6 February 2021) was used for image feature selection, model development, and performance assessment. R software package (R suite v3.6.2, https://www.r-project.org, 6 February 2021) was employed for other statistical tests. A two-sided *p*-value < 0.05 was considered significant.

## 3. Results

### 3.1. Demographics

Among the 168 candidate patients, 134 (aged 63.6 ± 8.9 years, 78 males and 56 females) were eligible for this study. Histological tests confirmed that all resected tumors were NSCLC, including 120 (89.6%) adenocarcinomas, 8 (6.0%) squamous cell carcinomas, 6 (4.4%) adenosquamous carcinomas. The NGS test determined 65 (48.5%), 15 (11.2%), 13 (9.7%), and 60 (44.8%) patients who had *EGFR, KRAS, ERBB2,* and *TP53* mutations, respectively. None of the patients had these four mutations at the same time. Moreover, 2 (1.5%), 39 (29.1%), and 69 (51.5%) patients had three, two, and one mutation, respectively, while 24 (17.9%) patients had no mutation. Table S2 shows the exon variants of these four mutations.

### 3.2. Extraction and Selection of Radiomic Features

The consistency analysis between the two feature extraction procedures showed that 1098 out of 1672 features were stable (Spearman’s *r* > 0.8) and usable for feature selection, including 199 first-order, 14 shape, and 885 texture features.

**Figure 3:**
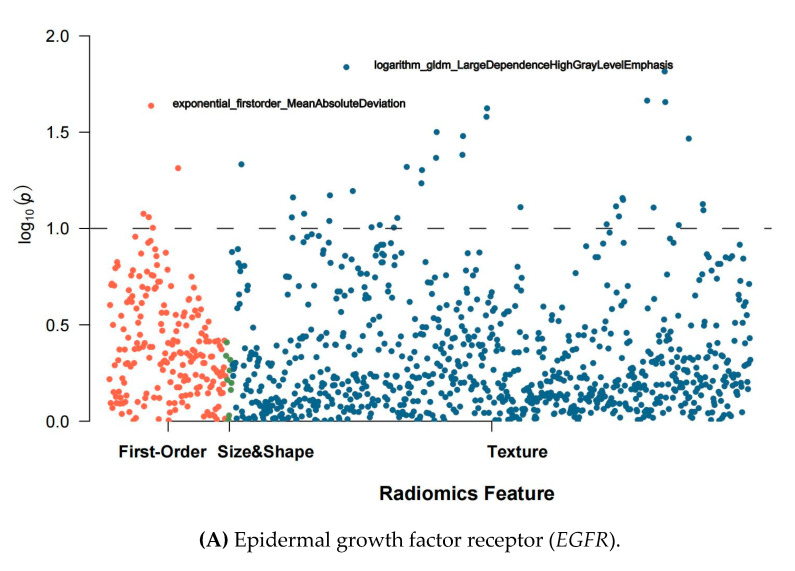

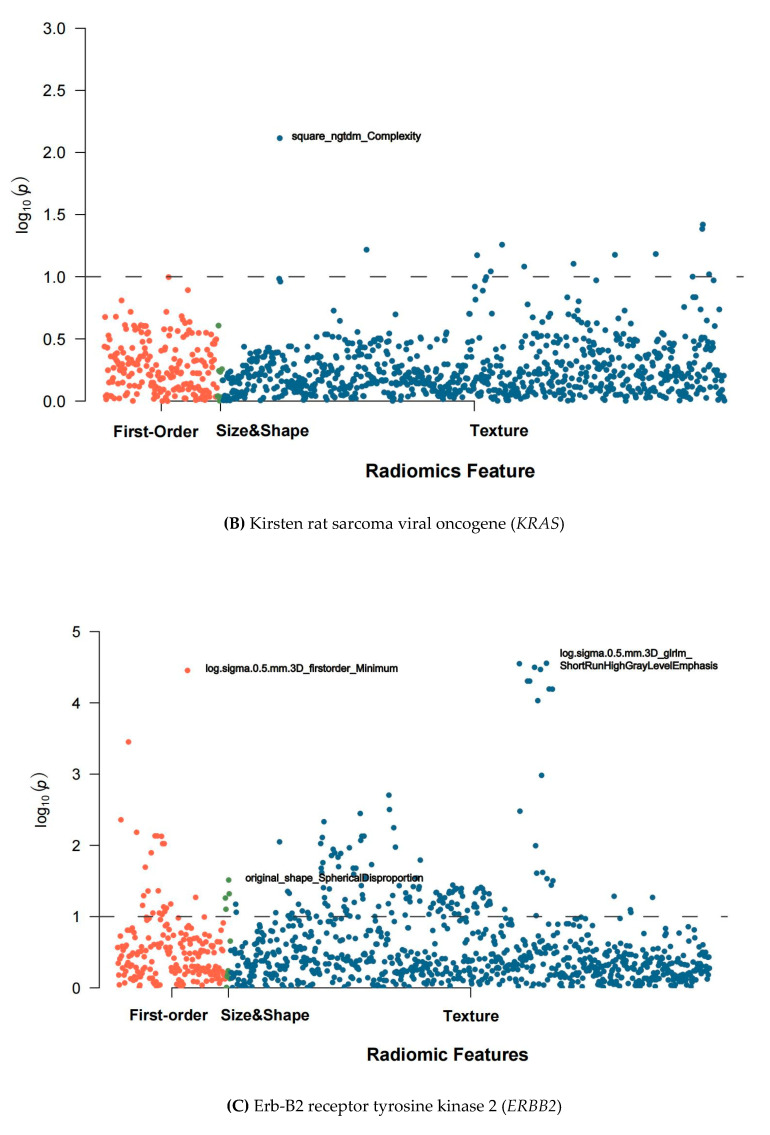

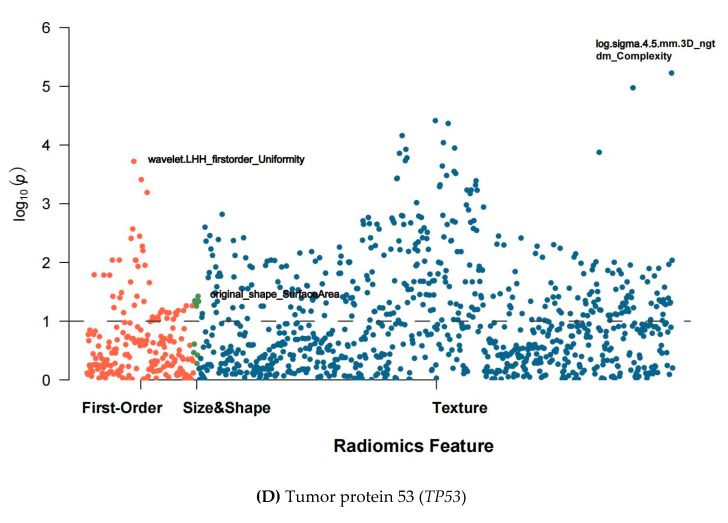
Manhattan plots of feature selection. The orange, green, and blue dots represent first-order, size&shape, and texture features, respectively. The features above the dashed line are those with a Spearman’s rank correlation coefficient >0.8, which are considered eligible for building discriminative models.

Among the 1098 usable features, 40, 13, 166, and 398 features were highly relevant (F-statistic test’s *p* > 0.1) to *EGFR*, *KRAS*, *ERBB2*, and *TP53* mutations, respectively (Figure 3). The 40 highly relevant features for *EGFR* mutation included five first-order and 35 texture features but did not include any size and shape-related features. The highest correlated first-order and texture features were exponential_firstorder_MeanAbsoluteDeviation and logarithm_gldm_LargeDependenceHighGray LevelEmphasis, respectively. The 13 highly relevant features for *KRAS* mutation were texture features, in which the highest correlated one was square_ngtdm_Complexity. The 166 highly relevant features for *ERBB2* included 23 first-order, 4 shape, and 139 texture features, in which the highest correlated features were log.sigma.0.5.mm.3D_firstorder_Minimum, original_shape_ SphericalDisproportion, and log.sigma.0.5.mm.3D_glrlm_ShortRunHighGrayLevelEmphasis, respectively. The 398 highly relevant features for *TP53* mutation included 43 first-order, six shape, and 349 texture features, in which wavelet.LHH_firstorder_Uniformity, original_shape_SurfaceArea, and log.sigma.4.5.mm.3D_ngtdm_ Complexity had the highest correlation, respectively.

Subsequently, the top 20 relevant features (13 for *KRAS*) in the F-statistic test to *EGFR*, *KRAS*, *ERBB2*, and *TP53* mutations were used to construct discriminative models. Detailed visualized results and distributions of these features are shown in Figures S1–S4. The finally selected features with a non-zero coefficient after LASSO selection for all mutations are presented in Table S3.

### 3.3. Model Performance

Gender was a significant factor associated with *EGFR* mutation (Wilcox rank-sum *p* = 0.001) and with *KRAS* mutation (*p* = 0.036). Tumor stage (cT) was a significant factor for *ERBB2* mutation (*p* = 0.044). Age, sex, and tumor metastasis (cM) were significant factors for *TP53* mutation (all *p* < 0.01). Then, these relevant clinical factors (Table S4) were combined with the above-mentioned radiomic features to establish combined models.

**Figure 4:**
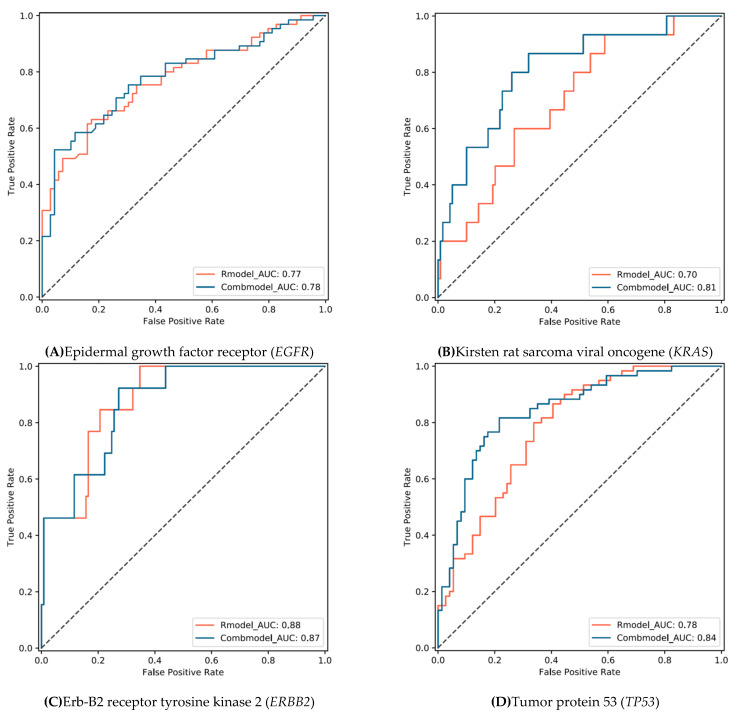
Receiver operating characteristic curves for discriminating mutations by the radiomics model and combined model (radiomic features and clinical factors).

The radiomics model and combined model showed similar performance in discriminating *EGFR* and *ERBB2* mutations. The AUC (Figure 4) of these two models for discriminating *EGFR* was 0.77 (95% CI: 0.70 to 0.85) and 0.78 (0.70 to 0.86), respectively (DeLong’s *p* = 0.590). The AUC of these two models for discriminating *ERBB2* was 0.88 (0.80–0.96) and 0.87 (0.78–0.95), respectively (*p* = 0.585). The combined model showed a sensitivity and specificity of 0.52 (0.40–0.65) and 0.96 (0.87–0.99) for discriminating *EGFR*, respectively (Table 1), and 0.92 (0.62–0.99) and 0.78 (0.67–0.87) for *ERBB2*, respectively.

**Figure 5:**
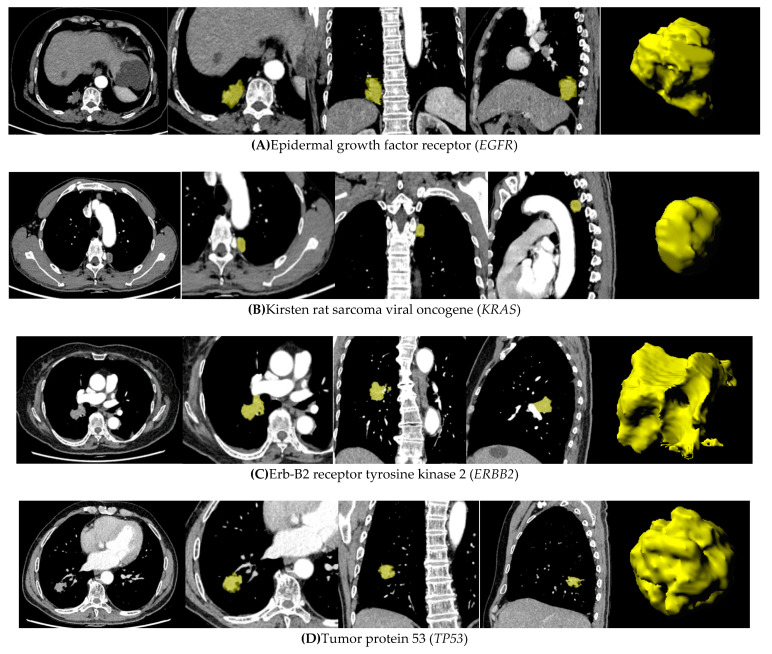
Representative CT images with tumor segmentation by a radiomics analysis platform. (**A**) A 30-year-old female non-smoker, with *EGFR* mutation in lung adenocarcinoma. A lobulated solid mass is observed in the lower lobe of the right lung. The maximum diameter is 23 mm. (**B**) A 64-year-old male smoker, with *KRAS* mutation in lung adenocarcinoma. A solid mass with rough margin is observed in the upper lobe of the left lung. The maximum diameter is 10 mm. (**C**) A 74 year-old female non-smoker, with *ERBB2* mutation in lung adenocarcinoma. A lobulated solid mass is observed in the middle lobe of the right lung. The maximum diameter is 18 mm. (**D**) A 66-year-old male smoker, with *TP53* mutation in lung adenocarcinoma. A lobulated solid mass with rough margin is observed in the lower lobe of the right lung. The maximum diameter is 12 mm.

The combined models showed improved performance in discriminating *KRAS* and *TP53* mutations. The combined model significantly improved the AUC of discriminating *KRAS* mutation from 0.70 (0.57–0.83) to 0.81 (0.69–0.93) (DeLong’s *p* = 0.044), and from 0.78 (0.71–0.86) to 0.84 (0.78–0.91) for *TP53* (*p* = 0.032). The combined model showed a sensitivity and specificity of 0.87 (0.58–0.97) and 0.68 (0.59–0.76) for discriminating *KRAS*, respectively, and 0.82 (0.69–0.90) and 0.78 (0.67–0.87) for *TP53*, respectively. Representative CT images with tumor segmentation are shown in Figure 5.

## 4. Discussion

In this study, we established machine learning-derived radiomics models to determine the presence of *EGFR*, *KRAS*, *ERBB2*, and *TP53* mutations in patients with NSCLC, based on radiomic features and combined with clinical factors. The AUC of the combined models ranged from 0.78 to 0.87 for discriminating these four mutations. In particular, the specificity to determine *EGFR* mutation was 0.96, indicating a very low false-positive rate that is potentially useful to screen outpatients with *EGFR* wildtype. The sensitivity to define *KRAS*, *ERBB2* and *TP53* mutations ranged from 0.82 to 0.92, suggesting a low false-negative rate, which is helpful in selecting patients with mutations for invasive sampling and NGS testing. Our study reveals the possibility of using a noninvasive method to screen for multiple genetic mutations before invasive sampling and expensive molecular testing.

The mutation status of *EGFR*, *KRAS*, *ERBB2,* and *TP53* is closely associated with the response to targeted therapy for NSCLC. *EGFR* is the main actionable target of many targeted therapies in patients with NSCLC [31]. *KRAS* mutation is also a common oncogenic driver [5]. Recently, novel therapeutic strategies for *KRAS* G12C, the most common *KRAS* mutation in NSCLC, have emerged [32,33]. A recent early-phase clinical trial evaluated the efficacy of zenocutuzumab, a bispecific *ERBB2*/*ERBB3* antibody [34]. *TP53* mutation is a potential negative prognostic factor for NSCLC patients with TKI therapy due to increased cellular resistance to *EGFR*-TKIs [10,35]. The simultaneous and rapid detection of these four mutations is crucial for clinical decision-making in patients with NSCLC.

Because lung cancer is a heterogeneous disease at the molecular level, testing for genetic alteration biomarkers has been recommended for each specimen of advanced-stage NSCLC [36]. NGS allows comprehensive polygenic analysis and facilitates the identification of alterations for targeted therapy. Before NGS, genomic analysis was limited to specific loci known to be associated with each cancer subtype. Single-gene sequencing like Sanger technology is limited to DNA insertion, deletion, and substitution, while NGS can detect chromosomal rearrangement, oncogenic fusion event, translocation, and copy number alteration. Therefore, this study took NGS as the reference standard. Although NGS is more cost-effective than multiple single-gene tests in detecting multiple genetic alterations, the cost of NGS is still high, which limits its clinical implementation. Our study demonstrated a noninvasive and low-cost method to screen patients with NSCLC before NGS testing. In particular, the high specificity (0.96) to determine *EGFR* mutation is potentially useful to screen outpatients with *EGFR* wildtype. The patients with negative radiomic results would have a high probability for wildtype, thus avoiding unnecessary NGS tests. The high sensitivity (0.82 to 0.92) to determine *KRAS*, *ERBB2*, and *TP53* mutations increases the certainty of detecting these mutations. Patients with positive radiomic results may have a higher probability of harboring mutations in these genes that could be validated through NGS.

Analysis of CT-based image features has received extensive attention on detecting *EGFR* mutation, limited attention to *KRAS* and *TP53* mutations, but no report on *ERBB2* mutation. In 385 patients with lung adenocarcinoma, Liu et al. found that using human semantic annotation of a CT scan combined with clinical variables reached an AUC of 0.78 to discriminate *EGFR*+/*EGFR*-, superior to using clinical variables alone (AUC = 0.69) [37]. Zhang et al. conducted a multivariate analysis based on CT radiomic features to discriminate *EGFR* mutation in patients with NSCLC and reached AUCs of 0.86 and 0.87 the training (*n* = 140) and test (*n* = 40) cohorts, respectively [38]. Recently, Wang et al. established a deep learning model to distinguish *EGFR*+/*EGFR*−, and reached AUCs of 0.85 and 0.81 in the training (*n* = 603) and test (*n* = 241) cohorts, respectively [39]. Our models achieved an AUC of 0.78 to identify *EGFR* mutation, which is comparable to the previous reports. However, there were few studies on discriminating *KRAS* and *TP53* mutations. Velazquez et al. developed radiomic signatures to distinguish *KRAS*+/*KRAS*−, *EGFR*+/*EGFR*−, and *EGFR*+/*KRAS*+ with a training cohort (*n* = 353) and reached AUCs of 0.63, 0.69, and 0.80 in an independent test cohort (*n* = 352), respectively [19]. Pinheiro et al. Included 116 and 114 patients with NSCLC to establish models to detect *EGFR* and *KRAS* mutations, respectively. They found that radiomic features were correlated with *EGFR* mutation (AUC = 0.58) but not *KRAS* (AUC = 0.51), and the semantic hybrid model improved the AUC to 0.74 for *EGFR* mutation status [22]. Wang et al. developed and validated a radiomics-based fusion-positive tumor prediction model in 61 patients with early-stage lung adenocarcinoma, which can discriminate *TP53*/*EGFR* mutations and tumor mutation burden, and yielded AUCs of 0.84 and 0.59 for identifying *TP53* mutation in the training (*n* = 41) and test cohorts (*n* = 20), respectively [9]. Our models achieved AUCs of 0.81 and 0.84 to identify *KRAS* and *TP53* mutations, respectively, which is higher than the previous reports.

This study’s major strength is to simultaneously analyze *EGFR*, *KRAS*, *ERBB2*, and *TP53* mutations in a single CT examination. The AUCs for discriminating these four mutations ranged from 0.78 to 0.88. The AUCs of *KRAS* and *TP53* were higher than the reported results (0.63 and 0.66, respectively) [9,19]. One reason to archive high AUCs might be a 3D radiomics algorithm, which was used in this study to extract and analyze 1672 radiomic features. The extracted features in most of the published radiomics studies were relatively fewer. We extracted 1672 radiomics features, which laid the foundation for machine learning to select highly relevant features. Thereafter, a wide range of candidate features can maximize the potential information hidden in the images, thus improving the capacity of reflecting the genotype of NSCLC lesions.

This study has some limitations. First, this retrospective study was conducted in one center. Ideally, a prospective multicenter study would enhance the conclusion of this study. The results may differ in case of the presence of mutations in other populations. Further research is necessary to test the generalizability of our models in other races. Second, we included 134 patients because the NGS test is expensive and not widely used in clinical practice. Increasing the sample size will strengthen the robustness of radiomic models. Third, the model described in this study has not been validated in an independent set. Forth, the extracted radiomic features can be prone to inter- and intra-observer variability as a consequence of the manual part of the image segmentation procedure.

## 5. Conclusion

Machine learning-derived 3D radiomics based on CT images can simultaneously identify the presence of *EGFR*, *KRAS*, *ERBB2*, and *TP53* mutations in patients with NSCLC, which can sensitively determine *EGFR* mutation with a very low false-positive rate, and increase the certainty of determining the presence of *KRAS*, *ERBB2*, and *TP53* mutations. These findings suggest that patients with a negative radiomics result of *EGFR* mutation can avoid expensive NGS testing, but patients with positive *KRAS*, *ERBB2*, and *TP53* results should undergo NGS testing. Although these conclusions should be validated in a larger sample size population, machine learning-derived radiomics has the potential to become a noninvasive and low-cost method to screen multiple genetic mutations in patients with NSCLC before using an NGS test, which can help improve individualized targeted therapy.

## Figures and Tables

**Table 1:** Summary of diagnostic metrics for discriminating *EGFR*, *KRAS*, *ERBB2*, and *TP53* mutations.

True Label		Radiomic Features						Combined Model (Radiomic Features and Clinical Factors)					
		Wildtype	Mutation	Sensitivity	Specificity	Accuracy	AUC	Wildtype	Mutation	Sensitivity	Specificity	Accuracy	AUC
*EGFR*	Wildtype (*n* = 69)	57	12	0.63(0.50–0.74)	0.83(0.71–0.90)	0.73(0.65–0.80)	0.77(0.70–0.85)	66	3	0.52(0.40–0.65)	0.96(0.87–0.99)	0.75(0.66–0.82)	0.78(0.70–0.86)
	Mutation (*n* = 65)	24	41					31	34				
*KRAS*	Wildtype (*n* = 119)	49	70	0.93(0.66–0.99)	0.41(0.32–0.51)	0.47(0.38–0.56)	0.70(0.57–0.83)	81	38	0.87(0.58–0.97)	0.68(0.59–0.76)	0.70(0.62- 0.78)	0.81(0.69–0.93)
	Mutation (*n* = 15)	1	14					2	13				
*ERBB2*	Wildtype (*n* = 121)	42	79	1.00(0.72–1.00)	0.65(0.56–0.74)	0.69(0.60–0.76)	0.88(0.80–0.96)	88	33	0.92(0.62–0.99)	0.73(0.64–0.80)	0.75(0.66–0.82)	0.87(0.78–0.95)
	Mutation (*n* = 13)	0	13					1	12				
*TP53*	Wildtype (*n* = 74)	49	25	0.80(0.67–0.89)	0.66(0.54–0.77)	0.72(0.64- 0.80)	0.78(0.71–0.86)	58	16	0.82(0.69–0.90)	0.78(0.67–0.87)	0.80(0.72–0.87)	0.84(0.78–0.91)
	Mutation (*n* = 60)	12	48					11	49				

*EGFR* = epidermal growth factor receptor; *KRAS* = Kirsten rat sarcoma viral oncogene; *ERBB2* = Erb-B2 receptor tyrosine kinase 2; *TP53* = tumor protein 53

## Data Availability

The data presented in this study are available on request from the corresponding author.

## Supplementary Materials

The following are available online at https://www.mdpi.com/article/10.3390/cancers13081814/s1, Figure S1: Logistic regression paths showing the coefficients of top 20 features (13 for *KRAS*) at different lambda values in the least absolute shrinkage and selection operator (LASSO) feature selection procedure, Figure S2, Correlation heatmaps with clustering of the most relevant radiomic features with the presence of genetic mutation, Figure S3, Heatmaps of the most relevant radiomic features with the presence of genetic mutation, Figure S4, Boxplots of the most relevant radiomic features with the presence of genetic mutation. Table S1: CT acquisition protocols and image reconstruction parameters, Table S2: Variation of *EGFR*, *KRAS*, *ERBB2*, and *TP53* mutations, Table S3: Finally selected features with non-zero coefficient after the least absolute shrinkage and selection operator (LASSO) selection, Table S4: Association between clinical factors and the presence of *EGFR*, *KRAS*, *ERBB2*, and *TP53* mutations. Radiomic feature interpretation.
