# Supplementary material for: Simultaneous Identification of EGFR, KRAS, ERBB2, and TP53 Mutations in Patients with Non-Small Cell Lung Cancer by Machine Learning-Derived Three-Dimensional Radiomics
Source: Cancers (Basel). 2021 Apr 10;13(8):1814. doi: 10.3390/cancers13081814 (PMC8070114; doi:10.3390/cancers13081814)
Supplement: Supplementary file 1 [file cancers-13-01814-s001.pdf]

Supple

Simultaneous identification of *EGFR, KRAS, ERBB2*, and *TP53* mutations in patients with non-small cell lung cancer by machine learning-derived three-dimensional radiomics

Tiening Zhang[1], Zhihan Xu[2], Guixue Liu[3], Beibei Jiang[3], Geertruida H. de Bock[4], Harry JM Groen[5], Rozemarijn Vliegenthart[6], Xueqian Xie[3]

Table S1. CT acquisition protocols and image reconstruction parameters

CT scanner	Siemens Somatom Force	GE Revolution
Acquisition mode	Helical	Helical
Tube voltage, kV	100	120
Tube current, mA	130 (quality reference)	100-400 (smart mode)
Collimation, mm	192×0.6	256×0.625
Pitch	1.2	0.984
Rotation time, ms	500	500
Reconstruction kernel	Br44	Standard
Field of view, mm	325	325
Slice thickness, mm	0.6	0.625
Slice increment, mm	0.6	0.625
Radiation dose, mGy	≈4.52	≈5.20

Table S2. Variation of *EGFR, KRAS, ERBB2*, and *TP53* mutations

EGFR		*KRAS*		*ERBB2*		*TP53*	
Mutation site	Number	Mutation site	Number	Mutation site	Number	Mutation site	Number
Exon-5	2	Exon-2	10	Exon-21	2	Exon-4	7
Exon-18	3	Exon-3	3	Exon-12	1	Exon-3	2
Exon-19	25	12P12.1	2	Exon-20	6	Exon-5	11
Exon-20	7			Exon-17	1	Exon-6	9
Exon-21	24			Exon-19	1	Exon-7	11
Exon18-25	1			Exon-22	1	Exon-8	12
7P11.2	3			17Q12	1	Exon-9	3
						C.1045G>T	1
						c.375+2T>A	1
						P.ARG248PRO	1
						C.602T>A	1
						P.LEU201*	1
Total	65	Total	15	Total	13	Total	60

EGFR= epidermal growth factor receptor; *KRAS*= Kirsten rat sarcoma viral oncogene; *ERBB2*= Erb-B2 receptor tyrosine kinase 2; *TP53*= tumor protein 53

EGFR Exon-5: p.A138V, p.V173M

Exon-18: p.G719A, p.E709A

Exon-19: C.2235_2249, C.2236_2250, C.2239_2255, C.2237_2255, C.2240_2254, C.2239_2248, p.E746_A750, p.L858R, p.E746_A750, p.E746_S752

Exon-20: p.N771_ p 772, p.T790M, p.S768_D770, p.H773_V774

Exon-21: p.L858R, p.L861Q, p.L858R

KRAS Exon-2: p.G12A, p.G12C, p.G12D, p.G12V, p.G13C

Exon-3: p.Q61H

ERBB2 Exon-21: p.L858R

Exon-12: C.1397C>T

Exon-20: C.2332, p.GLY778, C.2313, p.ALA775, p.Y772, p.G778,

Exon-17: p.V659E

Exon19: p.I767M

Exon22: p.R896G

TP53 Exon-3: C.96+1G>T,

Exon-4: p.S106Afs*17, p.E68Dfs*51, p.L43*, p.P72Rfs*41, C.375+1G>T, C.202G>T, p.GLU68*

Exon-5: p.E180K, p.H179R, p.V157F, p.R158L, p.V173L, p.H179R, p.Q167*, p.A159V, p.G154V, p.R158G

Exon-6: p.L194R , p.Y220S, p.E204*, p.H193Y, p.Q192*, p.R213L, p.S215G, p.V216M, C.569C>T, p.S241F, C.569C>T

Exon-7: p.R248G, p.G245S, p.R248W, C.673-2A>T, p.N235D, p.G244V, p.G244D, p.L257R, p.Y220C, p.M246V, C.736A>G, p.GLY245FS

Exon-8: p.I767M , p.C277F, P.R273C, p.R280K, p.GLU294FS, p.V272L, p.G266E, p.P278H, p.R280T, p.G266V

Exon-9: p.R896G, C.920-1G>A, p.E326Dfs*19

Table S3. Finally selected features with a non-zero coefficient after the least absolute shrinkage and selection operator (LASSO) selection

Model	EnrolledFeature	Feature Type	Spearman's correlation coefficient	Coefficient (LASSO Logistic)
EGFR	original_shape_Maximum2DDiameterSlice	Shape	0.992885	0.462044
	square_glcm_DifferenceAverage	Texture	0.88451	2.701276
	square_glcm_ JointEnergy	Texture	0.918446	-0.3922
	square_ngtdm_ Busyness	Texture	0.871921	0.127518
	square_ngtdm_Complexity	Texture	0.802408	-0.09055
	squareroot_firstorder_90Percentile	First-order	0.94855	2.658891
	squareroot_glrlm_ RunLengthNonUniformityNormalized	Texture	0.935961	-0.36438
	wavelet.LHH_glrlm_ShortRunEmphasis	Texture	0.819376	-0.44897
	wavelet.LHL_firstorder_10Percentile	First-order	0.901478	0.137372
	wavelet.LHL_firstorder_TotalEnergy	First-order	0.922824	-0.42447
	wavelet.LLH_firstorder_90Percentile	First-order	0.847291	-0.06422
	wavelet.LLH_glrlm_LongRunEmphasis	Texture	0.939245	-0.44264
	log.sigma.0.5.mm.3D_glszm_GrayLevelVariance	Texture	0.829228	-0.56618
ERBB2	original_glcm_Contrast	Texture	0.874658	-1.16287
	logarithm_glrlm_GrayLevelNonUniformity	Texture	0.996716	0.161683
	exponential_gldm_DependenceNonUniformity	Texture	0.992337	1.925881
	exponential_gldm_GrayLevelVariance	Texture	0.995751	0.360805
	exponential_ngtdm_Complexity	Texture	0.998938	-0.13601
	wavelet.LHH_gldm_DependenceNonUniformity	Texture	0.983032	-1.42815
	wavelet.LHL_gldm_GrayLevelVariance	Texture	0.827586	0.245614
	wavelet.LLH_glrlm_LongRunEmphasis	Texture	0.939245	-1.07933
	log.sigma.0.5.mm.3D_gldm_DependenceNonUniformityNormalized	Texture	0.918446	0.802951

	log.sigma.1.5.mm.3D_glrlm_GrayLevelVariance	Texture	0.893815	0.143292
	log.sigma.2.5.mm.3D_glcm_Imc1	Texture	0.975917	2.884485
	log.sigma.2.5.mm.3D_glcm_Imc2	Texture	0.896552	1.172461
	log.sigma.2.5.mm.3D_glszm_SizeZoneNonUniformity	Texture	0.95676	0.262752
	log.sigma.4.5.mm.3D_firstorder_Entropy	First-order	0.888889	-0.36531
	log.sigma.4.5.mm.3D_gldm_DependenceNonUniformity	Texture	0.992337	-0.36871
	log.sigma.4.5.mm.3D_ngtdm_Coarseness	Texture	0.992885	-2.55691
KRAS	squareroot_glcm_Idn	Texture	0.935961	0.595243
	wavelet.LHL_glcm_DifferenceVariance	Texture	0.841817	-0.02693
	wavelet.LLH_firstorder_RobustMeanAbsoluteDeviation	First-order	0.949097	-0.30775
	log.sigma.0.5.mm.3D_glcm_InverseVariance	Texture	0.914067	0.105427
	log.sigma.1.5.mm.3D_firstorder_Mean	First-order	0.993432	0.353714
	log.sigma.1.5.mm.3D_glrlm_RunVariance	Texture	0.992885	-0.065
	log.sigma.1.5.mm.3D_glszm_LargeAreaLowGrayLevelEmphasis	Texture	0.97318	-0.03965
	log.sigma.1.5.mm.3D_glszm_SizeZoneNonUniformity	Texture	0.955665	0.25912
	log.sigma.2.5.mm.3D_glcm_JointEntropy	Texture	0.966612	0.70026
	log.sigma.2.5.mm.3D_gldm_DependenceEntropy	Texture	0.915161	-0.23615
	log.sigma.3.5.mm.3D_firstorder_RobustMeanAbsoluteDeviation	First-order	0.840722	-0.07761
	log.sigma.3.5.mm.3D_glcm_ClusterShade	Texture	0.962233	0.315677
	log.sigma.3.5.mm.3D_gldm_DependenceVariance	Texture	0.984674	-0.8243
	log.sigma.4.5.mm.3D_glcm_ClusterProminence	Texture	0.872469	-0.53991
TP53	squareroot_glcm_Idn	Texture	0.935961	-0.7443
	wavelet.LHH_gldm_SmallDependenceEmphasis	Texture	0.83908	-0.49639
	wavelet.LHL_glcm_DifferenceEntropy	Texture	0.863164	-0.37893
	wavelet.LHL_glcm_DifferenceVariance	Texture	0.841817	0.10981
	wavelet.LLH_firstorder_RobustMeanAbsoluteDeviation	First-order	0.949097	-1.21147
	log.sigma.0.5.mm.3D_glcm_InverseVariance	Texture	0.914067	-0.89589
	log.sigma.1.5.mm.3D_firstorder_Mean	First-order	0.993432	-0.10977
	log.sigma.1.5.mm.3D_glszm_LargeAreaLowGrayLevelEmphasis	Texture	0.97318	-0.46992
	log.sigma.1.5.mm.3D_glszm_SizeZoneNonUniformity	Texture	0.955665	0.144011
	log.sigma.2.5.mm.3D_gldm_DependenceEntropy	Texture	0.915161	-0.191
	log.sigma.2.5.mm.3D_gldm_LowGrayLevelEmphasis	Texture	0.975917	-0.31674
	log.sigma.3.5.mm.3D_firstorder_RobustMeanAbsoluteDeviation	First-order	0.840722	0.026772
	log.sigma.3.5.mm.3D_glcm_ClusterShade	Texture	0.962233	0.102195

	log.sigma.3.5.mm.3D_gldm_DependenceVariance	Texture	0.984674	-0.38159
	log.sigma.3.5.mm.3D_glrlm_LongRunHighGrayLevelEmphasis	Texture	0.990148	0.478267
	log.sigma.3.5.mm.3D_glszm_LargeAreaHighGrayLevelEmphasis	Texture	0.992885	0.40209
	log.sigma.4.5.mm.3D_glcm_ClusterProminence	Texture	0.872469	-0.1152

Table S4. Association between clinical factors and the presence of *EGFR, KRAS, ERBB2, and TP53* mutations

	Total	*EGFR*			*KRAS*			*ERBB2*			*TP53*		
		Wildtype	Mutation	P Value	Wildtype	Mutation	P Value	Wildtype	Mutation	P Value	Wildtype	Mutation	P Value
Number	134	69	65		119	15		121	13		74	60	
Age, *years*	63.6±8.9	64.1±9.5	62.7±10.7	0.411	63.2±10.4	64.7±7.6	0.608	60.4±10.1	66.5±10.2	0.251	60.9±10.1	66.4±9.3	0.001*
Sex				0.001*			0.036*			0.221			0.008*
Female, ***n***	56	19	37		54	2		49	8		35	21	
Male, ***n***	78	50	28		65	13		73	5		39	39	
cT stage				0.813			0.602			0.044*			0.102
1, ***n***	35	17	18		30	5		32	3		23	12	
2, ***n***	42	24	18		37	5		40	2		22	20	
3, ***n***	26	12	14		25	1		25	1		17	9	
4, ***n***	31	16	15		27	4		24	7		12	19	
cN stage				0.873			0.850			0.956			0.336
0, *n*	26	13	13		24	2		23	3		18	8	
1, *n*	17	10	7		15	2		15	2		8	9	
2, *n*	65	34	31		58	7		59	6		36	29	
3, *n*	26	12	14		22	4		24	2		12	14	
cM stage				0.816			0.898			1.000			0.001*
0, *n*	56	30	26		49	7		51	5		21	35	
1, *n*	78	39	39		70	8		70	8		53	25	
Smoking status				0.190			0.357			0.877			0.206
Non-smoker, *n*	106	51	55		96	10		95	11		62	44	

Smoker, *n*	28	18	10		23	5		26	2		12	16	
Lesion location				0.270			0.362			0.485			0.210
Left upper lobe, *n*	36	21	15		29	7		33	3		24	12	
Left lower lobe, *n*	24	9	15		23	1		23	1		14	10	
Right upper lobe, *n*	39	23	16		36	3		35	4		21	18	
Right middle lobe, *n*	6	4	2		5	1		6	0		4	2	
Right lower lobe, *n*	29	12	17		26	3		24	5		11	18	

Note. P value indicates the significance between the wildtype and mutation by Wilcox rank sum test.

EGFR= epidermal growth factor receptor; *KRAS*= Kirsten rat sarcoma viral oncogene; *ERBB2*= Erb-B2 receptor tyrosine kinase 2; *TP53*= tumor protein 53.

Figure S1. Logistic regression paths showing the coefficients of top 20 features (13 for *KRAS*) at different lambda values in the least absolute shrinkage and selection operator (LASSO) feature selection procedure. The coefficient profile plots are preformed against the log(lambda) sequence. The dotted line shows the feature coefficient at the best lambda value for each mutation status. The coefficient of individual feature is represented by colored line.

A) Epidermal growth factor receptor (*EGFR*)

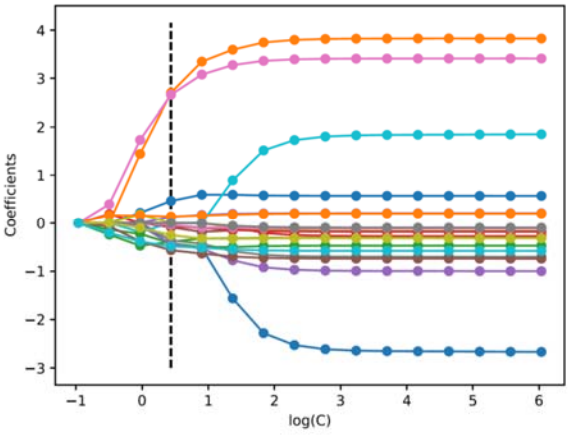

B) Kirsten rat sarcoma viral oncogene (*KRAS*)

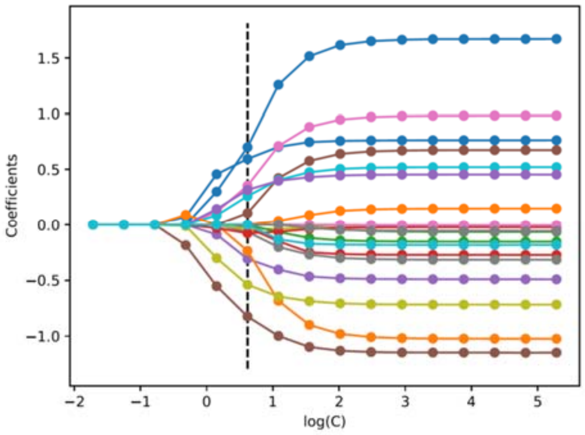

C) Erb-B2 receptor tyrosine kinase 2 (*ERBB2*)

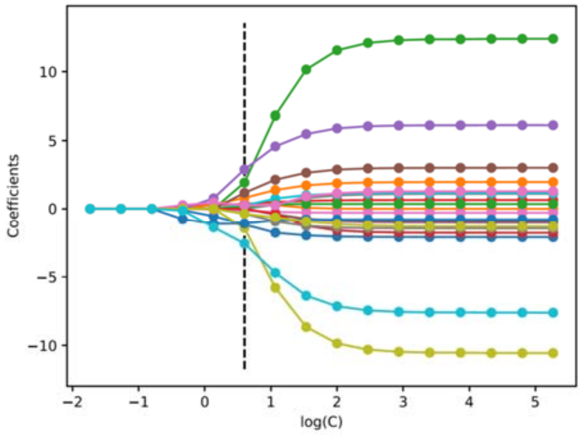

D) Tumor protein 53 (*TP53*)

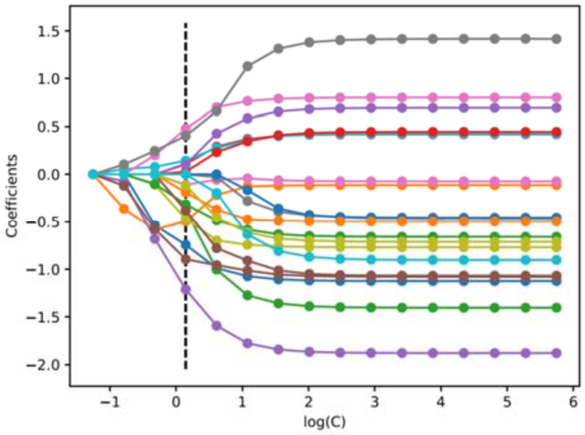

Figure S2. Correlation heatmaps with clustering of the most relevant radiomic features with the presence of genetic mutation. The color bar represents the Pearson's correlation coefficient (ϱ) between the features. The elements of the heatmap are color coded according to the Pearson's correlation coefficient between the features. Red color indicates that the two features are fully positive correlation, and blue color indicates complete negative correlation. The hierarchical cluster dendrogram is also shown in the figure. The top or bottom side of the correlation matrix square represents the most relevant features, and the feature from left to right horizontally is consistent with that from top to bottom vertically.

A) Epidermal growth factor receptor (*EGFR*)

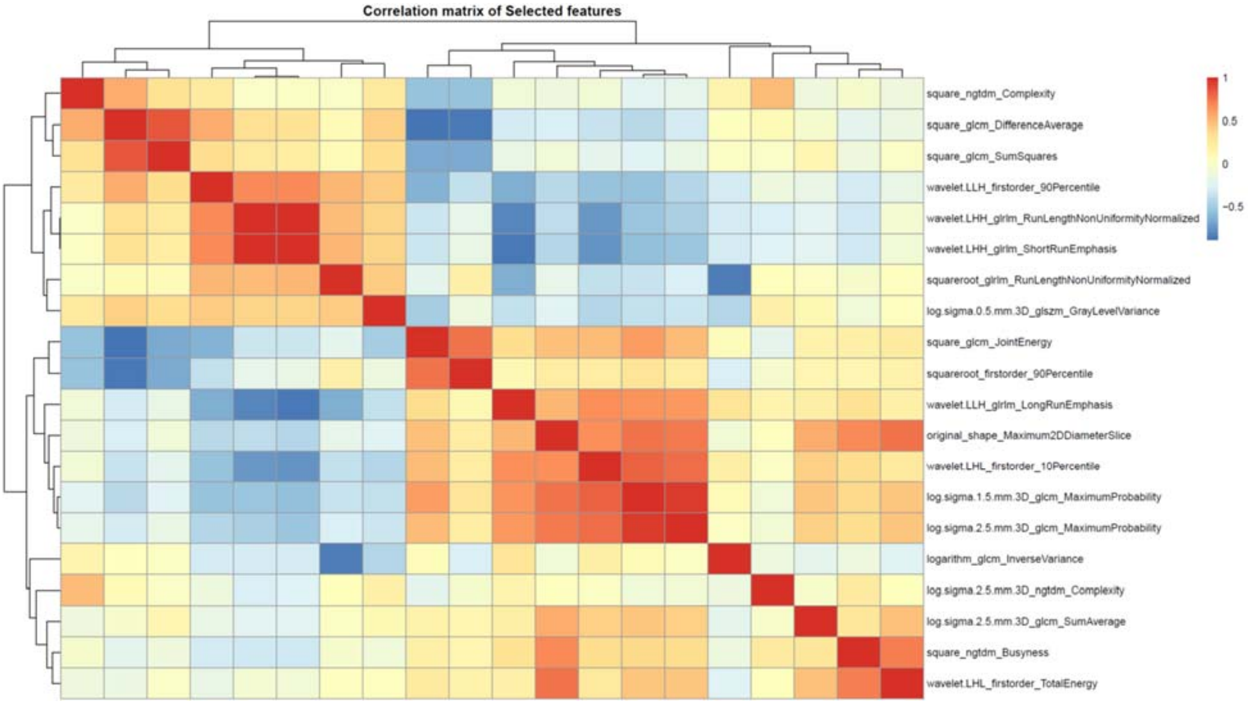

B) Kirsten rat sarcoma viral oncogene (*KRAS*)

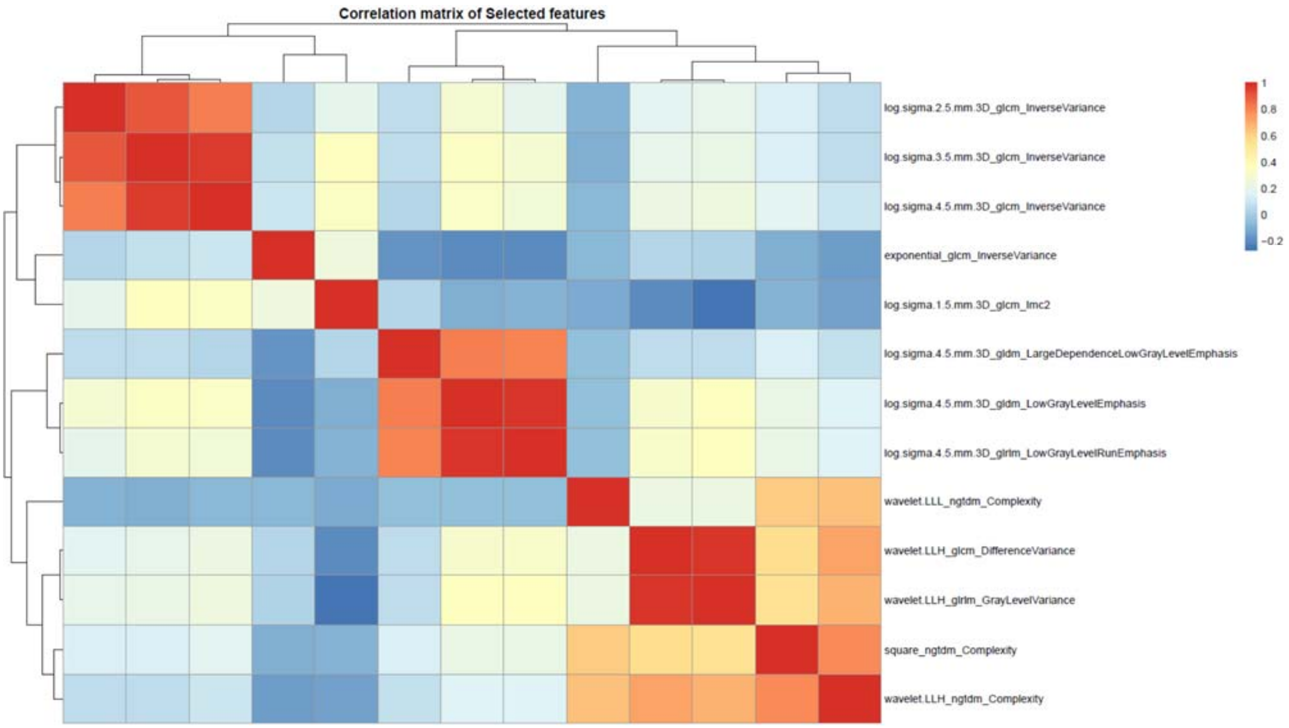

C) Erb-B2 receptor tyrosine kinase 2 (*ERBB2*)

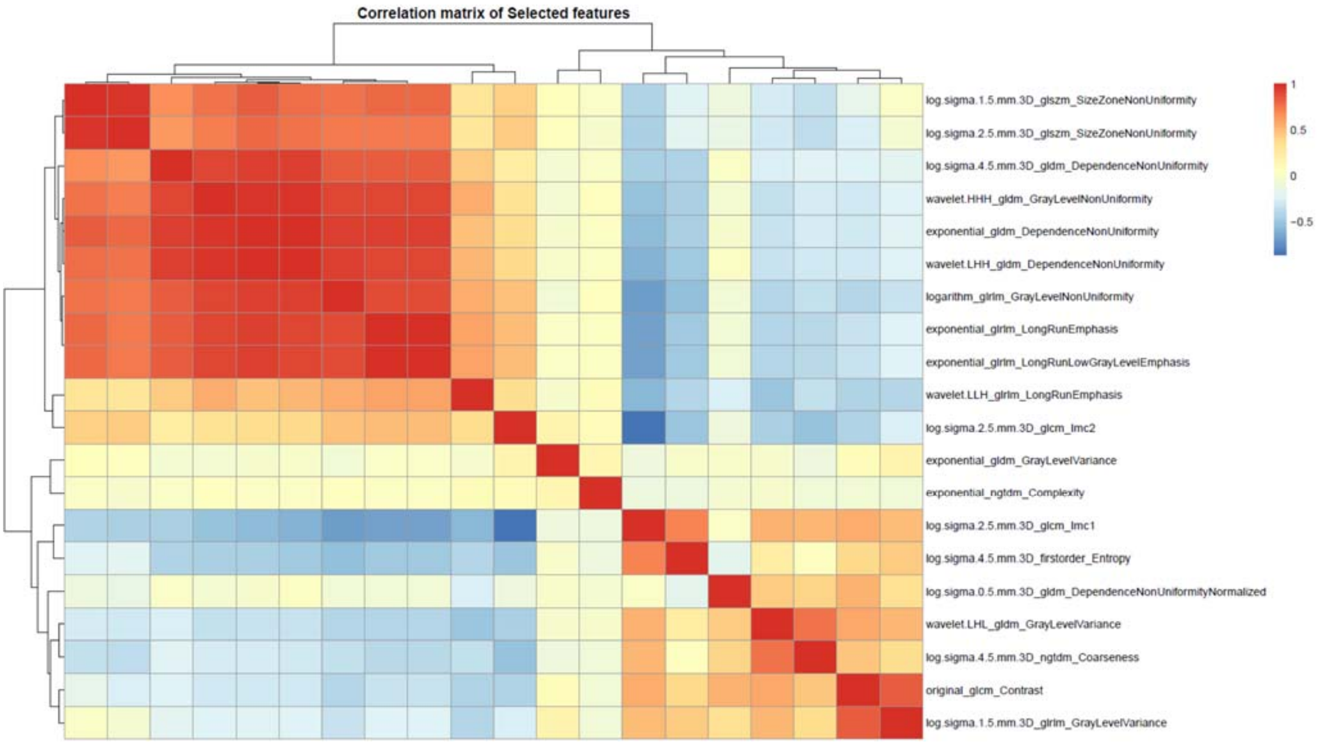

D) Tumor protein 53 (*TP53*)

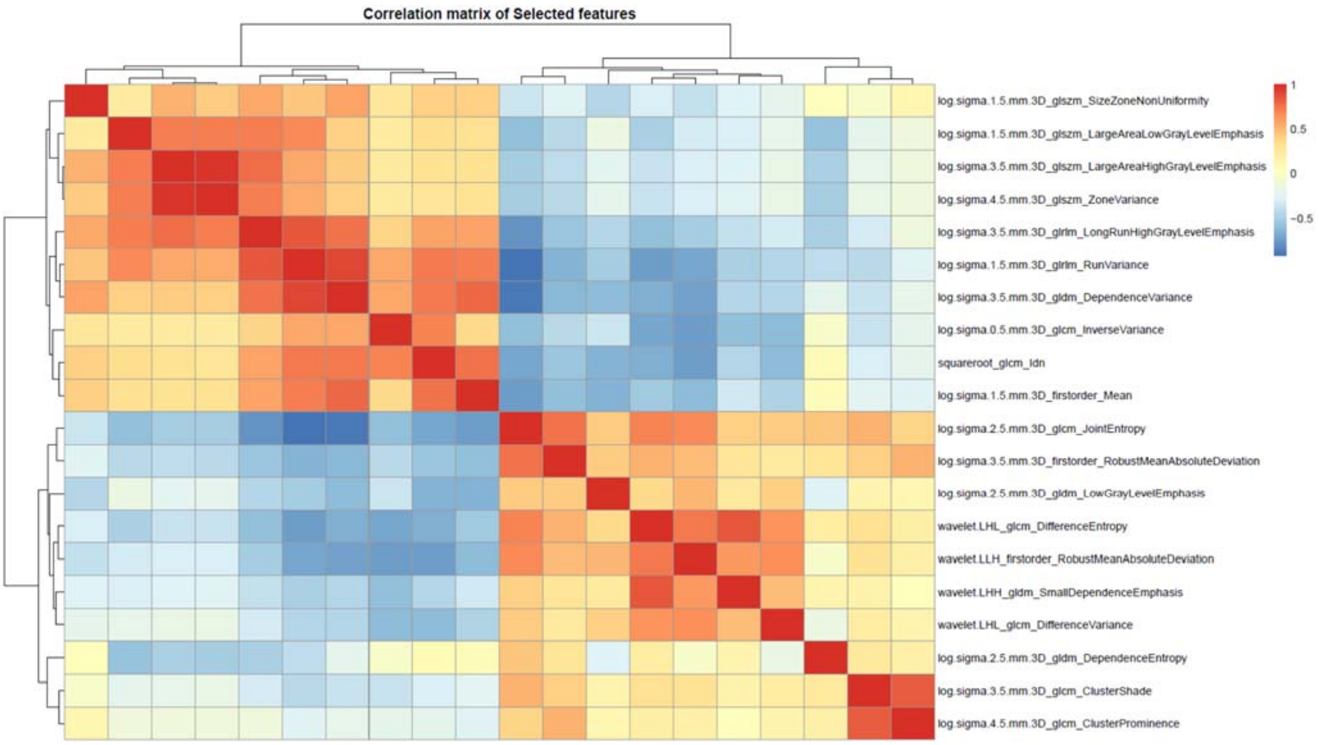
Correlation matrix of Selected features
log.sigma.1.5.mm.3D_glszm_SizeZoneNonUniformity
log.sigma.1.5.mm.3D_glszm_LargeAreaLowGrayLevelEmphasis
log.sigma.3.5.mm.3D_glszm_LargeAreaHighGrayLevelEmphasis
log.sigma.4.5.mm.3D_glszm_ZoneVariance
log.sigma.3.5.mm.3D_glrlm_LongRunHighGrayLevelEmphasis
log.sigma.1.5.mm.3D_glrlm_RunVariance
log.sigma.3.5.mm.3D_gldm_DependenceVariance
log.sigma.0.5.mm.3D_glcm_InverseVariance
squareroot_glcm_Idn
log.sigma.1.5.mm.3D_firstorder_Mean
log.sigma.2.5.mm.3D_glcm_JointEntropy
log.sigma.3.5.mm.3D_firstorder_RobustMeanAbsoluteDeviation
log.sigma.2.5.mm.3D_gldm_LowGrayLevelEmphasis
wavelet.LHL_glcm_DifferenceEntropy
wavelet.LLH_firstorder_RobustMeanAbsoluteDeviation
wavelet.LHH_gldm_SmallDependenceEmphasis
wavelet.LHL_glcm_DifferenceVariance
log.sigma.2.5.mm.3D_gldm_DependenceEntropy
log.sigma.3.5.mm.3D_glcm_ClusterShade
log.sigma.4.5.mm.3D_glcm_ClusterProminence
1
0.5
0
−0.5

Figure S3. Heatmaps of the most relevant radiomic features with the presence of genetic mutation. The color bar represents the normalized values for all features. The elements of the heat map are color coded according to the normalized feature values. Green color represents a larger value and yellow represents a smaller value.

A) Epidermal growth factor receptor (*EGFR*)

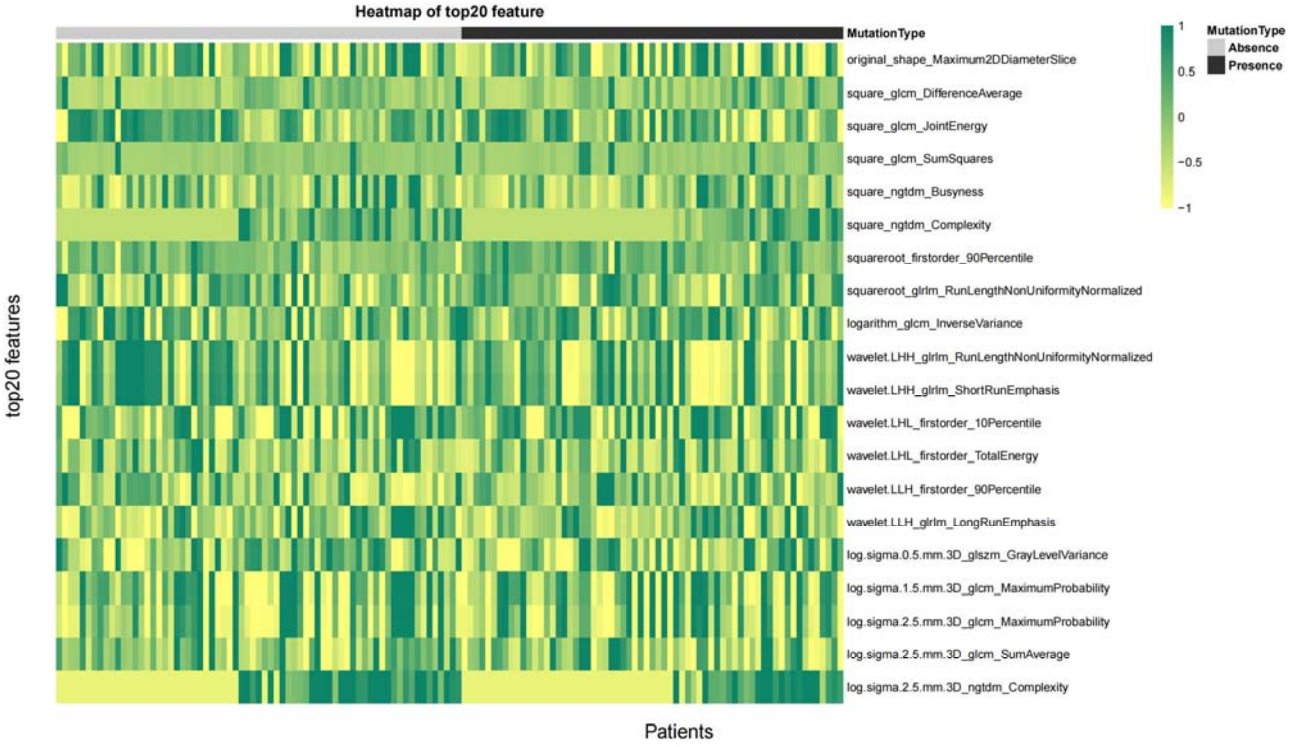

B) Kirsten rat sarcoma viral oncogene (*KRAS*)

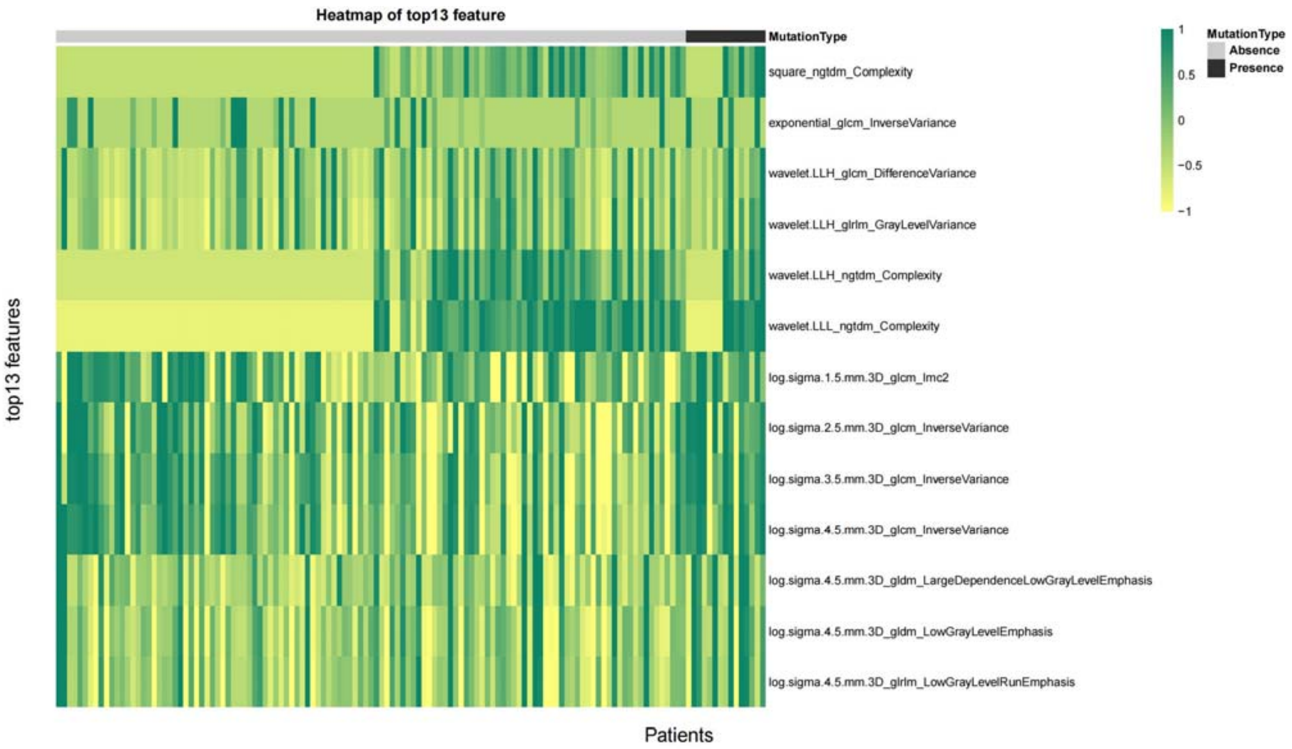

C) Erb-B2 receptor tyrosine kinase 2 (*ERBB2*)

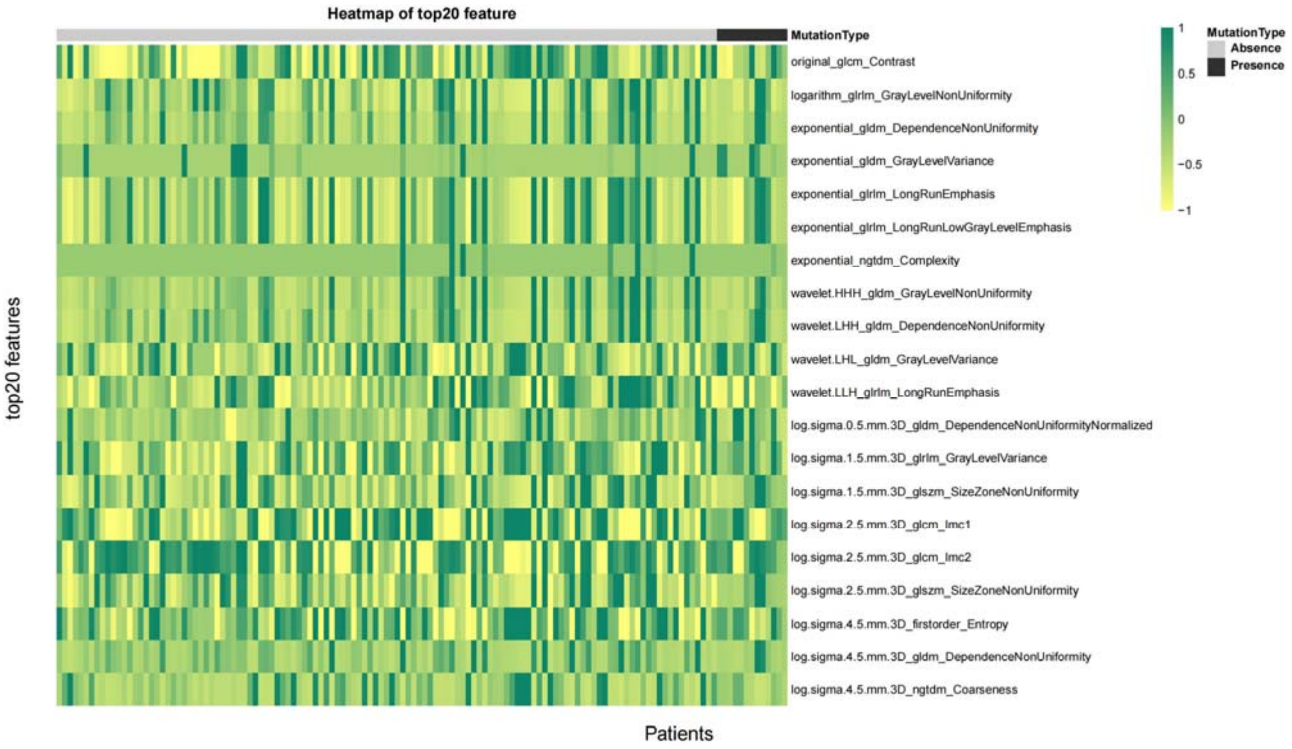

D) Tumor protein 53 (*TP53*)

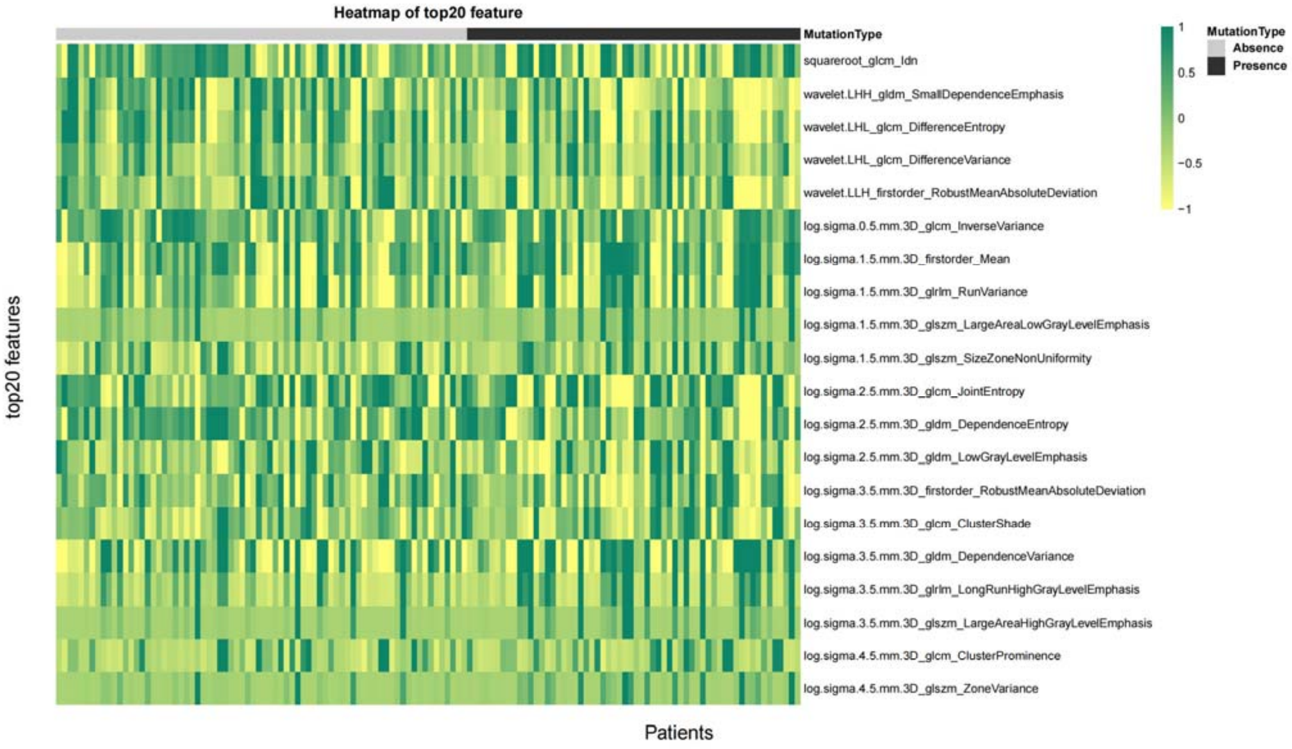

Figure S4. Boxplots of the most relevant radiomic features with the presence of genetic mutation

A) Epidermal growth factor receptor (*EGFR*)

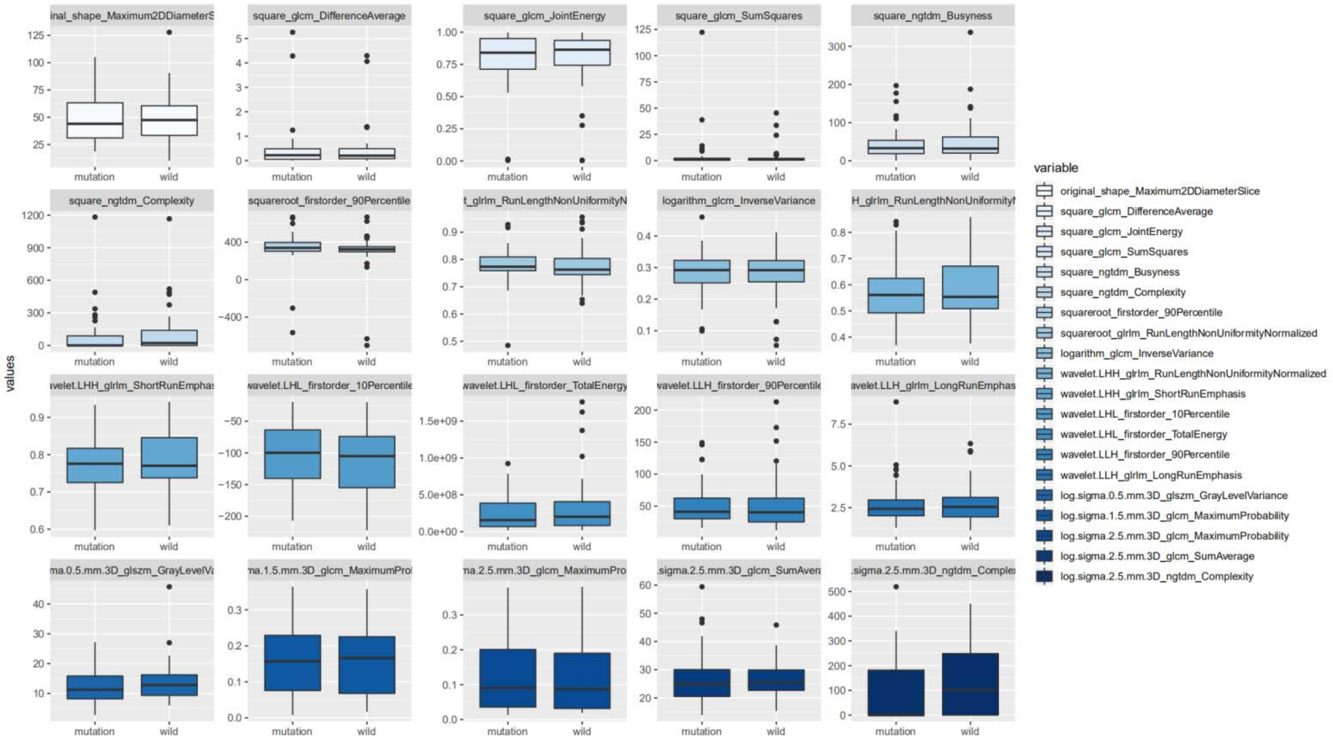

B) Kirsten rat sarcoma viral oncogene (*KRAS*)

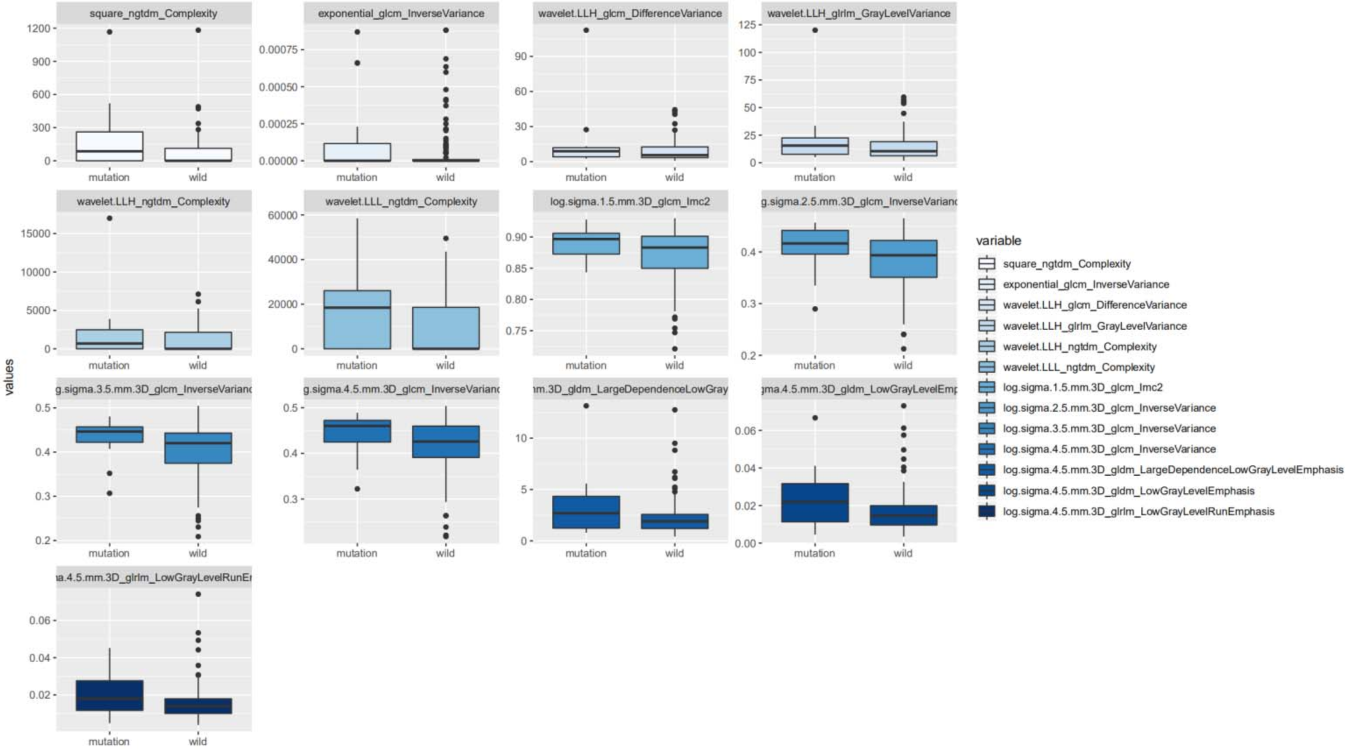

C) Erb-B2 receptor tyrosine kinase 2 (*ERBB2*)

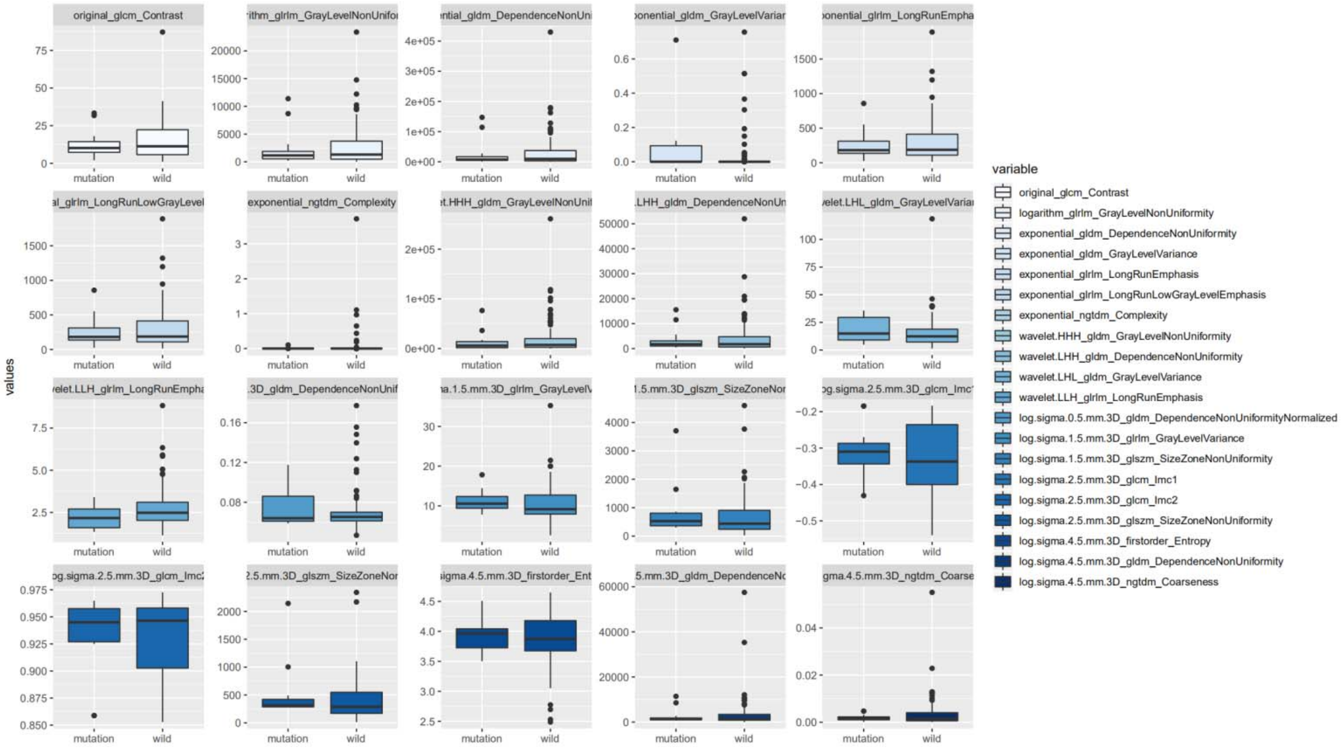

D) Tumor protein 53 (*TP53*)

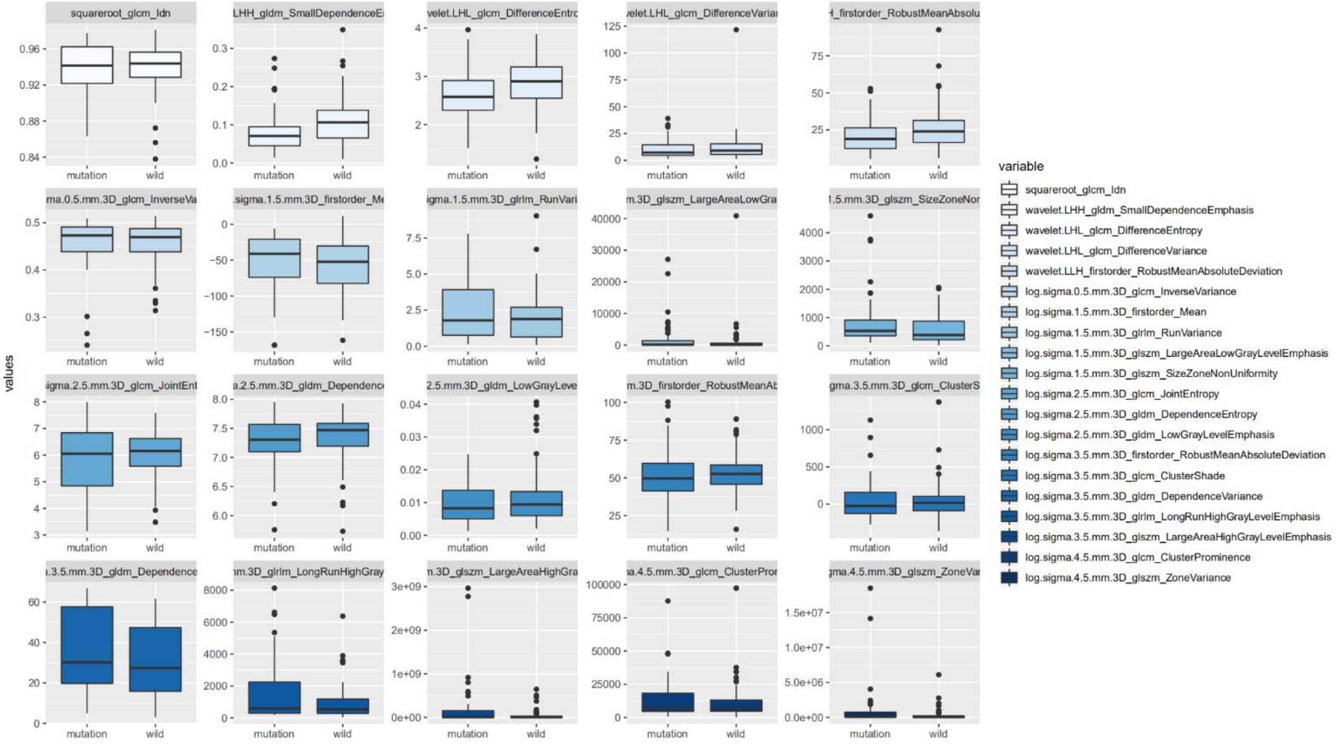

Supplemental materials

Radiomic feature interpretation:

1. original_shape_Maximum 2D Diameter Slice（*Maximum 2D diameter*）

Maximum diameter is defined as the largest pairwise Euclidean distance between tumor surface mesh vertices.

2. square_glcm_Difference Average

Difference average= $\sum_{k=0}^{N_g-1} kp_{x-y}(k)$

Difference Average measures the relationship between occurrences of pairs with similar intensity values and occurrences of pairs with differing intensity values.

3. square_glcm_Joint Energy

Joint energy= $\sum_{i=1}^{N_g}\sum_{j=1}^{N_g}(p(i,j))^2$

Energy is a measure of homogeneous patterns in the image. A greater Energy implies that there are more instances of intensity value pairs in the image that neighbor each other at higher frequencies.

4. square_glcm_Sum Squares

Sum squares= $\sum_{i=1}^{N_g}\sum_{j=1}^{N_g}(i-u_x)^2 p(i,j)$

Sum of Squares or Variance is a measure in the distribution of neigboring intensity level pairs about the mean intensity level in the GLCM.

5. square_ngtdm_Busyness

Busyness= $\frac{\sum_{i=1}^{N_g} p_i s_i}{\sum_{i=1}^{N_g}\sum_{j=1}^{N_g} |ip_i - jp_j|}$ *,where* $p_i \neq 0, p_j \neq 0$

A measure of the change from a pixel to its neighbour. A high value for busyness indicates a 'busy' image, with rapid changes of intensity between pixels and its neighbourhood.

N.B. if $N_{g,p} = 1$, then busyness= $\frac{0}{0}$. If this is the case, 0 is returned, as it concerns a fully homogeneous region.

6. square_ngtdm_Complexity*/log.sigma.2.5.mm.3D_ngtdm_Complexity/*exponential_ngtdm_Complexity

Complexity= $\frac{1}{N_{v,p}}\sum_{i=1}^{N_g}\sum_{j=1}^{N_g}|i-j|\frac{p_i s_i + p_j s_j}{p_i + p_j}$ *, where* $p_i \neq 0, p_j \neq 0$

An image is considered complex when there are many primitive components in the image, i.e. the image is non-uniform and there are many rapid changes in gray level intensity.

7. squareroot_firstorder_90Percentile/ wavelet.LLH_firstorder_90Percentile

The 9[0th] percentile of X

8. squareroot_glrlm_Run Length Non-Uniformity Normalized / wavelet.LHH_glrlm_Run Length Non-Uniformity Normalized (RLNN)

RLNN= $\frac{\sum_{j=1}^{N_r}(\sum_{i=1}^{N_g}P(i,j|\theta))^2}{N_r(\theta)^2}$

RLNN measures the similarity of run lengths throughout the image, with a lower value indicating more homogeneity among run lengths in the image. This is the normalized version of the RLN formula.

9. logarithm_glcm_Inverse Variance/log.sigma.0.5.mm.3D_glcm_InverseVariance

Inverse variance= $\sum_{k=1}^{N_g-1}\frac{p_{x-y}(k)}{\mathrm{k}^2}$

Note that $k=0$ is skipped, as this would result in a division by 0.

10. wavelet.LHH_glrlm_ShortRunEmphasis (SRE)

SRE= $\frac{\sum_{i=1}^{N_g}\sum_{j=1}^{N_r}\frac{P(i,j|\theta)}{j^2}}{N_r(\theta)}$

SRE is a measure of the distribution of short run lengths, with a greater value indicative of shorter run lengths and more fine textural textures.

11. wavelet.LHL_firstorder_10Percentile

The 10th percentile of X

12. wavelet.LHL_firstorder_Total Energy

total energy= $V_{voxel}\sum_{i=1}^{N_p}(X(i)+c)^2$

Here, c is optional value, defined by voxelArrayShift, which shifts the intensities to prevent negative values in X. This ensures that voxels with the lowest gray values contribute the least to Energy, instead of voxels with gray level intensity closest to 0.

Total Energy is the value of Energy feature scaled by the volume of the voxel in cubic mm.

***13. wavelet.LLH_glrlm_Long Run Emphasis/ exponential_glrlm_LongRunEmphasis** (LRE)*

LRE= $\frac{\sum_{i=1}^{N_g}\sum_{j=1}^{N_r} P(\mathrm{i}, j \mid \theta) j^2}{N_r(\theta)}$

LRE is a measure of the distribution of long run lengths, with a greater value indicative of longer run lengths and more coarse structural textures.

***14. log.sigma.0.5.mm.3D_glszm_GrayLevelVariance / exponential_gldm_GrayLevelVariance/ wavelet.LHL_gldm_GrayLevelVariance/ log.sigma.1.5.mm.3D_glrlm_GrayLevelVariance**(GLV)*

GLV= $\sum_{i=1}^{N_g}\sum_{j=1}^{N_s} p(i, j)(i-u)^2$

Here, $u = \sum_{i=1}^{N_g}\sum_{j=1}^{N_s} p(i, j) i$

GLV measures the variance in gray level intensities for the zones.

15. log.sigma.1.5.mm.3D_glcm_Maximum Probability/ log.sigma.2.5.mm.3D_glcm_MaximumProbability

maximum probability= $\max(p(i, j))$

Maximum Probability is occurrences of the most predominant pair of neighboring intensity values.

16. log.sigma.2.5.mm.3D_glcm_Sum Average

sum average= $\sum_{k=2}^{2N_g} p_{x+y}(k) k$

Sum Average measures the relationship between occurrences of pairs with lower intensity values and occurrences of pairs with higher intensity values.

17. original_glcm_Contrast

contrast= $\sum_{i=1}^{N_g}\sum_{j=1}^{N_g}(i-j)^2 p(i,j)$

Contrast is a measure of the local intensity variation, favoring values away from the diagonal $(i = j)$. A larger value correlates with a greater disparity in intensity values among neighboring voxels.

***18. logarithm_glrlm_Gray Level Non-Uniformity/ wavelet.HHH_gldm_Gray Level Non-Uniformity** (GLN)*

GLN= $$\frac{\sum_{i=1}^{N_g}(\sum_{j=1}^{N_r}P(\mathrm{i},j\,|\,\theta))^2}{N_r(\theta)}$$

GLN measures the similarity of gray-level intensity values in the image, where a lower GLN value correlates with a greater similarity in intensity values.

19. exponential_gldm_Dependence Non-Uniformity/wavelet.LHH_gldm_Dependence Non-Uniformity/log.sigma.4.5.mm.3D_gldm_Dependence Non-Uniformity

(DN)

DN= $$\frac{\sum_{j=1}^{N_d}(\sum_{i=1}^{N_g}P(i,j))^2}{N_z}$$

Measures the similarity of dependence throughout the image, with a lower value indicating more homogeneity among dependencies in the image.

***20. exponential_glrlm_LongRunLowGrayLevelEmphasis** (LRLGLE)*

LRLGLE= $$\frac{\sum_{i=1}^{N_g}\sum_{j=1}^{N_\mathrm{r}}\frac{P(i,j\,|\,\theta)j^2}{i^2}}{N_r(\theta)}$$

LRLGLRE measures the joint distribution of long run lengths with lower gray-level values.

***21. log.sigma.0.5.mm.3D_gldm_Dependence Non-Uniformity Normalized** (DNN)*

DNN= $$\frac{\sum_{j=1}^{N_\mathrm{d}}(\sum_{i=1}^{N_g}P(i,j))^2}{N_\mathrm{z}^2}$$

Measures the similarity of dependence throughout the image, with a lower value indicating more homogeneity among dependencies in the image. This is the normalized version of the DLN formula.

22. *log.sigma.1.5.mm.3D_glszm_Size-Zone Non-Uniformity / log.sigma.2.5.mm.3D_glszm_Size-Zone Non-Uniformity (SZN)*

$$SZN = \frac{\sum_{j=1}^{N_s}\left(\sum_{i=1}^{N_g} P(i,j)\right)^2}{N_z}$$

SZN measures the variability of size zone volumes in the image, with a lower value indicating more homogeneity in size zone volumes.

23. *log.sigma.2.5.mm.3D_glcm_Imc1(Informational Measure of Correlation 1)*

$$IMC1 = \frac{HXY - HXY1}{\max\{HX, HY\}}$$

IMC1 assesses the correlation between the probability distributions of ii and jj (quantifying the complexity of the texture), using mutual information I(x, y):

$$I(i,j) = \sum_{i=1}^{N_g}\sum_{j=1}^{N_g} p(i,j)\log_2\left(\frac{p(i,j)}{p_x(i)p_y(j)}\right)$$
$$= \sum_{i=1}^{N_g}\sum_{j=1}^{N_g} p(i,j)(\log_2(p(i,j)) - \log_2(p_x(i)p_y(j)))$$
$$= \sum_{i=1}^{N_g}\sum_{j=1}^{N_g} p(i,j)\log_2(p(i,j)) - \sum_{i=1}^{N_g}\sum_{j=1}^{N_g} p(i,j)\log_2(p_x(i)p_y(j))$$
$$= -HXY + HXY1$$

However, in this formula, the numerator is defined as HXY - HXY1 (i.e. −I(x,y)), and is therefore ≤0. This reflects how this feature is defined in the original Haralick paper.

In the case where the distributions are independent, there is no mutual information and the result will therefore be 0.

In the case of uniform distribution with complete dependence, mutual information will be equal to $\log_2(N_g)$.

Finally, HXY−HXY1 is divided by the maximum of the 2 marginal entropies, where in the latter case of complete dependence (not necessarily uniform; low complexity) it will result in IMC1=–1, as HX=HY=I(i,j).

24. *log.sigma.2.5.mm.3D_glcm_Imc2(Informational Measure of Correlation 2)*

$$IMC2 = \sqrt{1 - e^{-2(HXY2 - HXY)}}$$

IMC2 also assesses the correlation between the probability distributions of i and j (quantifying the complexity of the texture). Of interest is to note that HXY1=HXY2 and that HXY2−HXY≥0 represents the mutual information of the 2

distributions. Therefore, the range of IMC2 = [0, 1), with 0 representing the case of 2 independent distributions (no mutual information) and the maximum value representing the case of 2 fully dependent and uniform distributions (maximal mutual information, equal to $\log_2(N_g)$) . In this latter case, the maximum value is then equal to $\sqrt{1-e^{-2\log_2(N_g)}}$, approaching 1.

25. log.sigma.4.5.mm.3D_firstorder_Entropy

$$Entropy=-\sum_{i=1}^{N_g} p(i)\log_2(p(i)+\varepsilon)$$

Here, ϵ is an arbitrarily small positive number ($\approx 2.2\times 10^{-16}$).

Entropy specifies the uncertainty/randomness in the image values. It measures the average amount of information required to encode the image values.

26. log.sigma.4.5.mm.3D_ngtdm_Coarseness

$$Coarseness=\frac{1}{\sum_{i=1}^{N_g} p_i s_i}$$

Coarseness is a measure of average difference between the center voxel and its neighbourhood and is an indication of the spatial rate of change. A higher value indicates a lower spatial change rate and a locally more uniform texture.

N.B. $\sum_{i=1}^{N_g} p_i s_i$ potentially evaluates to 0 (in case of a completely homogeneous image). If this is the case, an arbitrary value of 10^6 is returned.

27. squareroot_glcm_Idn(Inverse Difference Normalized)

$$IDN=\sum_{k=0}^{N_g-1}\frac{p_{x-y}(k)}{1+(\frac{k}{N_g})}$$

IDN (inverse difference normalized) is another measure of the local homogeneity of an image. Unlike Homogeneity1, IDN normalizes the difference between the neighboring intensity values by dividing over the total number of discrete intensity values.

28. wavelet.LHH_gldm_SmallDependenceEmphasis(SDE)

$$SDE = \frac{\sum_{i=1}^{N_g}\sum_{j=1}^{N_d}\frac{P(i,j)}{i^2}}{N_z}$$

A measure of the distribution of large dependencies, with a greater value indicative of larger dependence and more homogeneous textures.

29. wavelet.LHL_glcm_Difference Entropy

$$\textit{Difference entropy} = \sum_{k=0}^{N_g-1} p_{x-y}(k)\log_2(p_{x-y}(k)+\varepsilon)$$

Difference Entropy is a measure of the randomness/variability in neighborhood intensity value differences.

30. wavelet.LHL_glcm_Difference Variance

$$\textit{difference Variance} = \sum_{k=0}^{N_g-1}(k-DA)^2 \mathrm{p}_{x-y}(k)$$

Difference Variance is a measure of heterogeneity that places higher weights on differing intensity level pairs that deviate more from the mean.

31. wavelet.LLH_firstorder_Robust Mean Absolute Deviation/ log.sigma.3.5.mm.3D_firstorder_RobustMeanAbsoluteDeviation (rMAD)

$$\mathrm{r}MAD = \frac{1}{N_{10-90}}\sum_{i=1}^{N_{10-90}}|X_{10-90}(i)-\overline{X}_{10-90}|$$

Robust Mean Absolute Deviation is the mean distance of all intensity values from the Mean Value calculated on the subset of image array with gray levels in between, or equal to the 10th and 90th percentile.

32. log.sigma.1.5.mm.3D_firstorder_Mean

$$\mathrm{m}ean = \frac{1}{N_p}\sum_{i=1}^{N_p} X(i)$$

The average gray level intensity within the ROI.

33. log.sigma.1.5.mm.3D_glrlm_RunVariance（RV)

$$RV = \sum_{i=1}^{N_g}\sum_{j=1}^{N_r} p(i,j|\theta)(j-u)^2$$

Here, $u=\sum_{i=1}^{N_g}\sum_{j=1}^{N_r}p(i,j|\theta)j$

RV is a measure of the variance in runs for the run lengths.

34. log.sigma.1.5.mm.3D_glszm_Large Area Low Gray Level Emphasis (LALGLE)

$$LALGLE=\frac{\sum_{i=1}^{N_g}\sum_{j=1}^{N_s}\frac{P(i,j)j^2}{i^2}}{N_z}$$

LALGLE measures the proportion in the image of the joint distribution of larger size zones with lower gray-level values.

35. log.sigma.2.5.mm.3D_glcm_Joint Entropy

$$\text{joint entropy}=-\sum_{i=1}^{N_g}\sum_{j=1}^{N_g}p(i,j)\log_2(p(i,j)+\varepsilon)$$

Joint entropy is a measure of the randomness/variability in neighborhood intensity values.

36. log.sigma.2.5.mm.3D_gldm_Dependence Entropy(DE)

$$\text{dependence entropy}=-\sum_{i=1}^{N_g}\sum_{j=1}^{N_d}p(i,j)\log_2(p(i,j)+\varepsilon)$$

37. log.sigma.2.5.mm.3D_gldm_LowGrayLevelEmphasis (LGLE)

$$LGLE=\frac{\sum_{i=1}^{N_g}\sum_{j=1}^{N_d}\frac{P(i,j)}{i^2}}{N_z}$$

Measures the distribution of low gray-level values, with a higher value indicating a greater concentration of low gray-level values in the image.

38. log.sigma.3.5.mm.3D_glcm_Cluster Shade

$$\text{Cluster Shade}=\sum_{i=1}^{N_g}\sum_{j=1}^{N_g}(i+j-u_x-u_y)^3p(i,j)$$

Cluster Shade is a measure of the skewness and uniformity of the GLCM. A higher cluster shade implies greater asymmetry about the mean.

39. log.sigma.3.5.mm.3D_gldm_Dependence Variance(DV)

$$DV = \sum_{i=1}^{N_g}\sum_{j=1}^{N_d} p(i,j)(j-u)^2 \ , \ where\ u = \sum_{i=1}^{N_g}\sum_{j=1}^{N_d} jp(i,j)$$

Measures the variance in dependence size in the image.

40. log.sigma.3.5.mm.3D_glrlm_Long Run High Gray Level Emphasis(LRHGLE)

$$LRHGLRE = \frac{\sum_{i=1}^{N_g}\sum_{j=1}^{N_r} P(i,j|\theta) i^2 j^2}{N_r(\theta)}$$

LRHGLRE measures the joint distribution of long run lengths with higher gray-level values.

41. log.sigma.3.5.mm.3D_glszm_Large Area High Gray Level Emphasis(LAHGLE)

$$LAHGLE = \frac{\sum_{i=1}^{N_g}\sum_{j=1}^{N_s} P(i,j) i^2 j^2}{N_z}$$

LAHGLE measures the proportion in the image of the joint distribution of larger size zones with higher gray-level values.

42. log.sigma.4.5.mm.3D_glcm_Cluster Prominence

$$Cluster\ Prominence = \sum_{i=1}^{N_g}\sum_{j=1}^{N_g} (i + j - u_x - u_y)^4 p(i,j)$$

Cluster Shade is a measure of the skewness and uniformity of the GLCM. A higher cluster shade implies greater asymmetry about the mean.

43. log.sigma.4.5.mm.3D_glszm_Zone Variance (ZV)

$$ZV = \sum_{i=1}^{N_g}\sum_{j=1}^{N_s} p(i,j)(j-u)^2$$

Here, $u = \sum_{i=1}^{N_g}\sum_{j=1}^{N_s} p(i,j)j$

ZV measures the variance in zone size volumes for the zones.

Biological meaning of the most-relevant features with mutations

The radiomic features with the lowest p values associated with *EGFR*, *KRAS*, *ERBB2* and *TP53* mutations are listed as follows:

1. EGFR

a) exponential_firstorder_MeanAbsoluteDeviation (mean distance of all intensity values from the mean value of the image array) indicates the dispersion of CT values in the volume of interest (VOI), which may be associated with the heterogeneity of tumor tissue.

b) logarithm_gldm_LargeDependenceHighGray LevelEmphasis (measures the joint distribution of large dependence with higher gray-level values) expresses the distribution of high CT value part, which may be associated with the vascularization of tumor.

2. *ERBB2*

a) square_ngtdm_Complexity considers an image as complex when there are many primitive components in the image, i.e., the image is non-uniform and there are many rapid changes in gray level intensity. This feature may be associated with the heterogeneity of tumor tissue.

3. *KRAS*

a) log.sigma.0.5.mm.3D_firstorder_Minimum indicates the minimum value in VOI, which may be associated with the heterogeneity of tumor tissue.

b) original_shape_SphericalDisproportion. Spherical Disproportion is the ratio of the surface area of the tumor region to the surface area of a sphere with the same volume as the tumor region, and by definition, the inverse of Sphericity. Therefore, the value range is spherical disproportion ≥1, with a value of 1 indicating a perfect sphere. This feature may associated with the irregular shaped tumor.

c) log.sigma.0.5.mm.3D_glrlm_ShortRunHighGrayLevelEmphasis indicates the homogeneity of high CT values, which may associated with the homogeneity of contrast-enhanced tumor tissues.

4. *TP53*

a) wavelet.LHH_firstorder_Uniformity indicates homogeneity, which may associated with the homogeneity of tumor tissue.

b) original_shape_SurfaceArea indicates surface area, which may associated with tumor size.

c) log.sigma.4.5.mm.3D_ngtdm_Complexity also considers an image as complex when there are many primitive components in the image, i.e., the image is non-uniform and there are many rapid changes in gray level intensity. This feature may be associated with the heterogeneity of tumor tissue.
